# Tomographic Study of Mesopore Formation in Ceria Nanorods

**DOI:** 10.1021/acs.jpcc.1c01221

**Published:** 2021-05-03

**Authors:** C. Brambila, D. C. Sayle, M. Molinari, J. Nutter, J. M. Flitcroft, T. X. T. Sayle, T. Sakthivel, S. Seal, G. Möbus

**Affiliations:** † Department of Materials Science and Engineering, University of Sheffield, Sheffield S1 3JD, U.K.; ‡ School of Physical Sciences, University of Kent, Canterbury CT2 7NZ, U.K.; § Department of Chemistry, University of Huddersfield, Huddersfield HD1 3DH, U.K.; ∥ Advanced Materials Processing and Analysis Center, Nanoscience and Technology Center (NSTC), Mechanical, Materials and Aerospace Engineering (MMAE), College of Medicine, Biionix Cluster, University of Central Florida, Orlando, Florida 32816, United States; ⊥ The Henry Royce Institute, Sir Robert Hadfield Building, Sheffield S1 3JD, U.K.

## Abstract

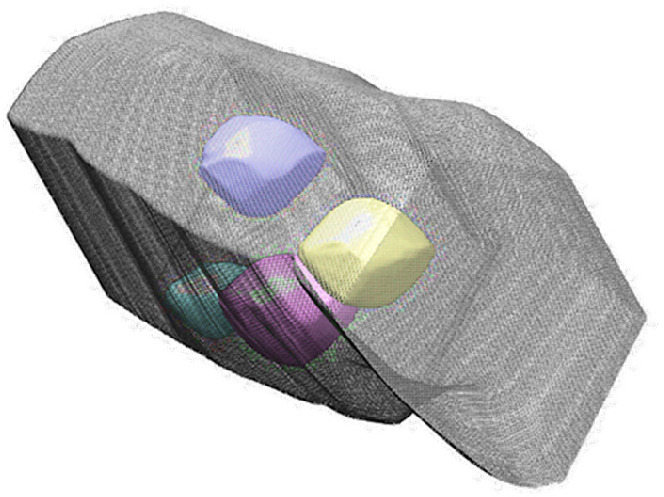

Porosity in functional oxide nanorods is a recently discovered new type of microstructure, which is not yet fully understood and still under evaluation for its impact on applications in catalysis and gas/ion storage. Here we explore the shape and distribution of pores in ceria in three dimensions using a modified algorithm of geometric tomography as a reliable tool for reconstructing defective and strained nanoobjects. The pores are confirmed as “negative-particle” or “inverse-particle” cuboctahedral shapes located exclusively beneath the flat surface of the rods separated via a sub-5 nm thin ceria wall from the outside. New findings also comprise elongated “negative-rod” defects, seen as embryonic nanotubes, and pores in cube-shaped ceria. Furthermore, we report near-sintering secondary heat treatment of nanorods and cubes, confirming persistence of pores beyond external surface rounding. We support our experiments with molecular modeling and predict that the growth history of voids is via diffusion and aggregation of atomic point defects. In addition, we use density functional theory to show that the relative stability of pore (shape) increases in the order “cuboidal” < “hexagonal-prismatic” < “octahedral”. The results indicate that by engineering voids into nanorods, via a high-temperature postsynthetic heat treatment, a potential future alternative route of tuning catalytic activities might become possible.

## Introduction

Ceria has remained one of the most studied nanostructured materials for many decades.^1^ Its abundance and versatility have seen its impact increase steadily. Since 2014, over half of the publications on nanoceria have concerned uses in catalysis.^2^ However, cutting-edge applications can be found across many other disciplines from biomedical^3^ to energy and environmental^4,5^ processes. The high redox activity and structural stability of ceria-based materials make them particularly appealing for the ever-growing field of heterogeneous catalysis, where novel structures are constantly being developed and tested for their catalytic performance.^6^ Rod-shaped nanoparticles are of particular interest, as they present higher activity than other ceria nanostructures of similar surface area.^7−9^ This is attributed to the presence of exposed planes and to the number of {100} and {110} surfaces.

More recently, there have been reports of porosity in ceria nanorods, at first for micropores (<2 nm) of nonspecific shape randomly found as part of the synthesis process.^10,11^ A hydrothermal process with systematic postengineering of pores via heat treatment has been found leading to larger well-faceted pores.^12,13^ Similar mesopores have been further elaborated and found to enhance the already high catalytic performance of ceria nanorods.^14,15^ Electron microscopy has so far been mostly used for 2D structural imaging of nanoceria, for example,^13,16^ and in few cases for electron tomography of ceria particles.^17,18^ While the tomography of solid and microporous rods^10^ is also available, there is no exploration into the three-dimensional shape and distribution of the newer heat-induced mesopore defects in the shape of “negative particles”. We believe that such a study is essential to understand the growth history and benefits the design and applications of such sophisticatedly engineered mesoporous ceria nanorods.

**Figure 1:**
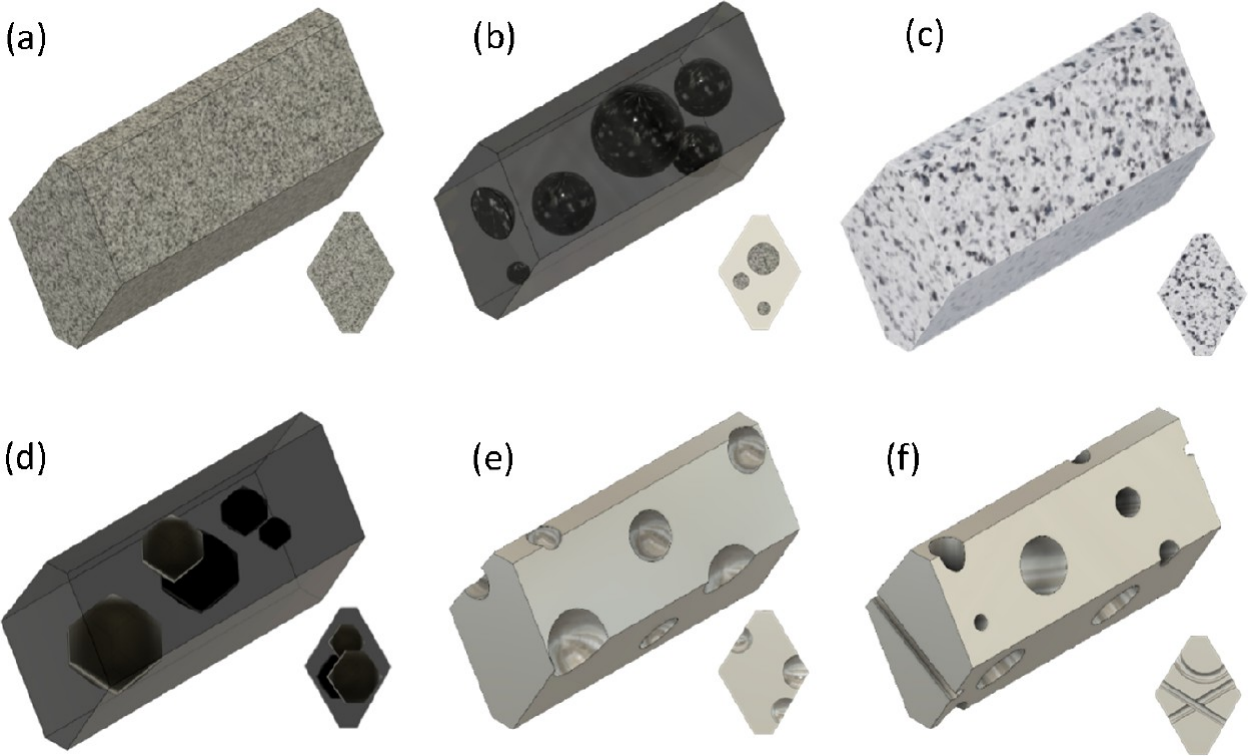
Schematic representation of different propositions of porous structures: (a) point defects/vacancies, (b) vacancy clusters, (c) microvoids, (d) mesopores with “negative particle” facet shape, (e) surface pores, etch pits, and concavities, and (f) through holes, nanochannels, and wormholes.

Figure 1 shows a schematic classification of different 3D microstructures that could correspond to images of defective nanorods commonly known. This list is by no means exhaustive and only aims to present some of the most likely possible origins for these features. We distinguish (a) patches of high concentrations of any point defects (vacancies and interstitials), possibly combined with lattice swelling;^19^ (b) agglomeration of these defects into clusters of ordered and neighboring point defects without forming voids;^11,20^ and (c) micropores of sub-2 nm sized voids of irregular shape randomly distributed over all the volume.^10,13,21,22^ (d) corresponds to internal mesopores of 2–10 nm size with well-faceted geometry, often termed “negative particles”, which are the topic of the present work;^13,23,24,28^ (e) refers to surface pores (indents, recesses, and etch pits);^20,25−27^ and (f) refers to through pores (nanochannels and wormhole shapes).^14,29,30^ While cases (a, b) are commonly found as result of irradiation experiments, (c–f) more commonly stem from chemical processes, including etching, phase separation, reactions, and heat treatments.

Some of those pores, for example, Figure 1e, have been excellently resolved by characterization.^27^ However, when TEM instead of AFM is needed, it becomes particularly relevant to note that several of these proposed porous structures could appear very similar in conventional 2D projection imaging; therefore, tomographic examination appears as the essential way forward to resolve the microstructure origin. Here we introduce a modified version of geometric tomography^32−34^ applied to electron tomography of crystalline materials with nonconvex features, not normally eligible for this technique. These explorations continue the work presented by Sakthivel et al.,^13^ which established reproducible annealing methods for the production of defect patterns in CeO_2_ nanorods. We seek to resolve the unanswered questions regarding the structure and morphology of those defects. We also expand the earlier work by a comparison of rod- and cube-shaped nanostructures and by increasing the range of heat treatment right up to the sintering starting temperature.

## Methods

### Materials Preparation

The detailed synthesis of our samples has been previously published by Sakthivel el al.^12,13^ In brief, different morphologies (including rod, cube, and octahedral particles) of CeO_2_ were achieved by controlling parameters (temperature and aging time) in a hydrothermal synthesis using Ce(NO_3_)_3_·6H_2_O as the ceria precursor and NaOH as reaction agent. Postsynthesis heat treatment was mostly conducted by conventional furnace heating of powders; however, we also added to the procedure some heat treatment of small powder samples suspended on Si/Si_3_N_4_ TEM carrier films, annealed in air with a heating rate of 5 °C/min, held at 800 °C for 3 h, and allowed to cool in the furnace overnight. The second temperature of 950 °C involved the same heating rate with 1 h holding time before cooling overnight. This latter procedure allows for TEM samples of specific powders to be viewed before and after heat treatment without disturbing intermediate specimen preparation.

### Materials Characterization

Apart from the mentioned Si_3_N_4_ specimens, typical powdered samples were suspended in deionized water and mounted onto standard copper grids with continuous or holey carbon film. High-resolution structural analysis was performed by using a JEOL JEM-F200 and a JEOL JEM-2010F field emission transmission electron microscope. Electron tomography or 3D-TEM was performed by using a JEOL JEM-3010 coupled with a Gatan model 912 tomography holder. Tilt series were aligned by using TomoJ.^48^ Then, they were segmented and binarized to create paths that precisely tracked the features of interest. The binary images were processed via Back-Projection algorithm, also in TomoJ. Finally, the stacks were rendered by using Chimera^49^ to image and measure the three-dimensional reconstructions.

### DFT Methodology

DFT calculations were performed by using the VASP 5 code.^50,51^ Simulations used a plane-wave basis set, which incorporates relativistic effect core potentials and the projector augmented wave (PAW) method. The exchange-correlation functional applied was the Perdew–Burke–Ernzerhof PBE. The Hubbard *U* correction, using the Dudarev methodology, was 5 eV.^52,53^ The cutoff energy of the plane-wave basis set was 500 eV. The electronic and ionic convergence criteria were 1 × 10^–4^ eV atom^–1^ and 1 × 10^–2^ eV Å^–1^. The Brillouin zone was sampled by using the Γ point. The energy of the void was calculated by removing the energy of a stoichiometric cubic unit cell comprising 256 CeO_2_ from the energy of the structure containing the void and the energy corresponding to the nanoparticle that was carved out to leave the void behind (9, 11, and 8 CeO_2_ units for the cube-like, hexagon-like, and octahedron-like shaped voids).

### Classical Molecular Dynamics Methodology

Molecular dynamics (MD) simulations were performed by using the DL_POLY code^54^ using the Born model of the ionic solid. The energy of the system is given by
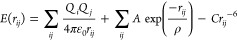
1where the first term represents the Coulombic interaction between ions *i* and *j* of charge *Q*_*i*_ and *Q*_*j*_ at a distance of *r*_*ij*_. The second term represents the Buckingham form, and potential parameters were taken from ref (55). A rigid ion model representation was used to reduce computational cost. Model ceria nanorod structures were generated by using simulated crystallization, following procedures reported previously.^44,56−59^ Graphical analysis of the molecular dynamical trajectories was performed by using Visual Molecular Dynamics (VMD).^60^ Calculation of the Madelung energies was performed by using the METADISE code.^61^

## Results

### Overview by Two-Dimensional Imaging

**Figure 2:**
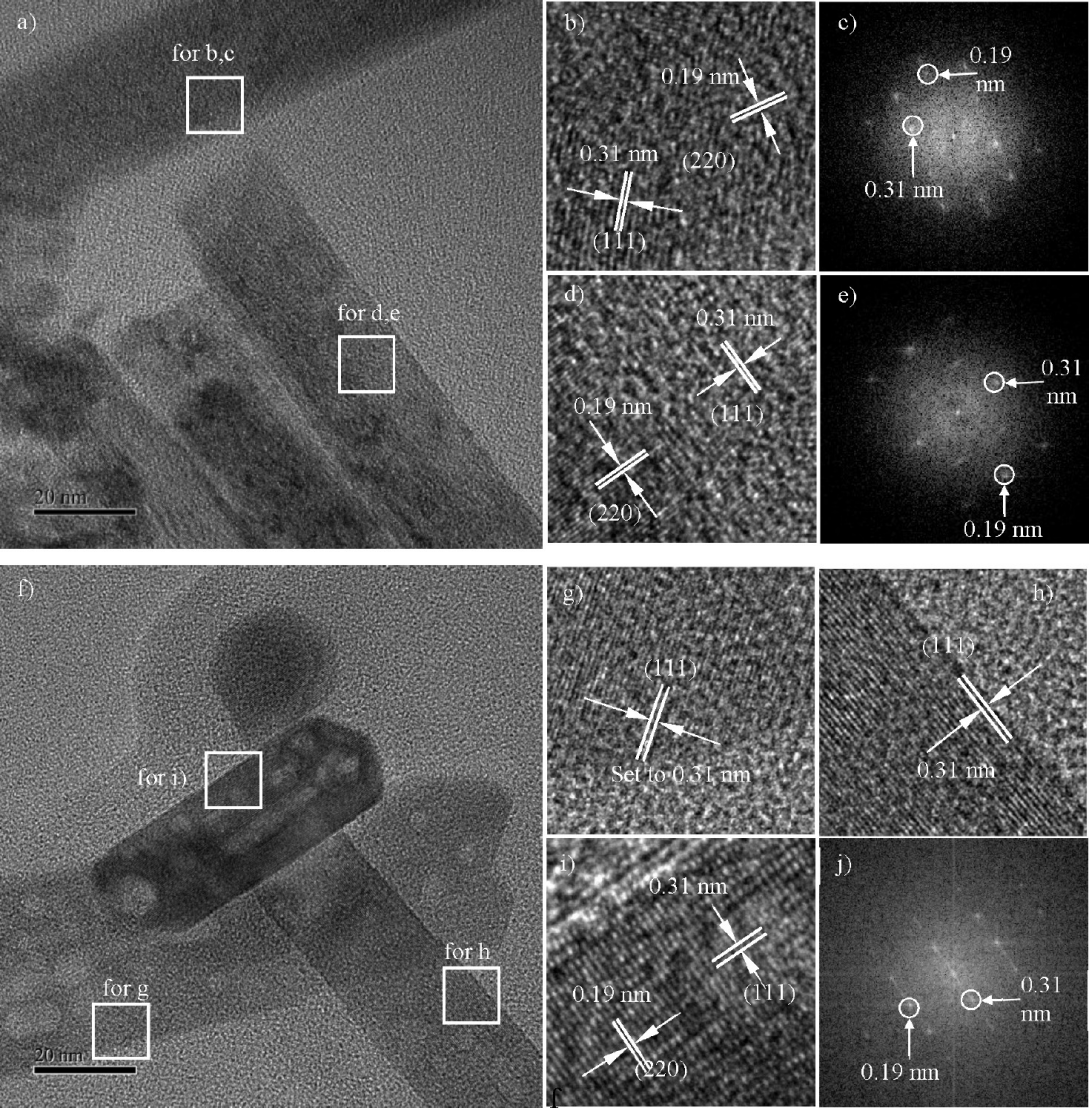
Crystallography of ceria nanorods. (a) Before heat treatment; with insets b and d for magnified lattice planes and insets c and e for FFT diffraction analysis; segment b is viewed near ⟨110⟩ on a ⟨110⟩ rod axis, while segment d is mainly a ⟨211⟩ rod axis, although the FFT indicates a superposition of two local grains. (f) After heat treatment; with insets g, h, and i for magnified lattice planes and inset j for FFT diffraction analysis. Segment g has a ⟨110⟩ rod axis and is viewed near ⟨110⟩, and segment i has a ⟨211⟩ rod axis and is viewed along ⟨110⟩, while segment h is near ⟨110⟩.

At first, ceria nanorod samples before and after heat treatment in air at 800 °C (mounted onto carbon-coated copper grid) are presented for overview in Figure 2. The nonheated rods show overall single crystallinity and homogeneity on the mesoscale (>2 nm) but a high density of sub-2 nm features, such as strain, lattice imperfections, and micropores (Figure 2a). In contrast, after heating, the microimperfections appear swapped against new mesoscale pores, with the overall lattice now healed to greater sub-2 nm perfection (Figure 2f).

The three neighboring rods visible represent a typical diversity, with aspect ratios varying significantly, for example, diameters ranging from 20 to 45 nm and lengths from 50 up to 200 nm. The rods show a continuous single-crystalline cubic structure with one of two growth directions: the rods highlighted (h) and (i) exhibit growth in the [211] direction while nanorod (g) has [110] growth direction, consistent with cited work.^35,36^ Both types of rods would show a nonregular hexagon cross section, enclosed by {111}/{110} facets for the ⟨211⟩ rod axis or {100}/{110} facets for the ⟨110⟩ axis.

**Figure 3:**
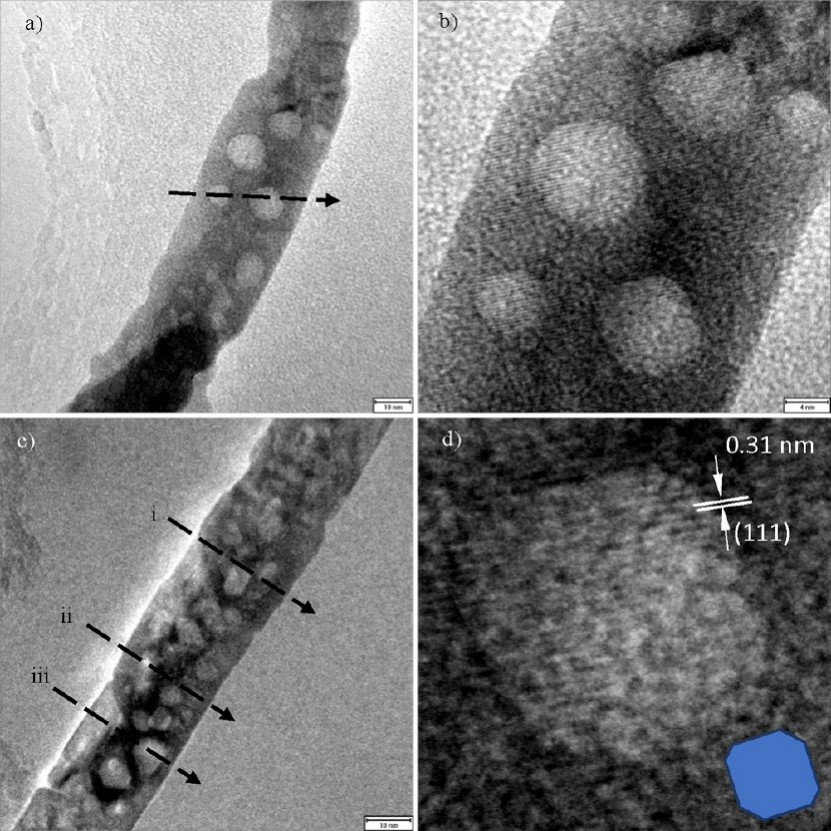
(a) Bright-field TEM image of a ceria nanorod with ⟨110⟩ axis, annealed at 800 °C. (b) HRTEM imaging of the same region showing lattice fringes and clear facets parallel to the {111} plane orientation. (c) 25 nm nanorod also with ⟨110⟩ axis, used for profile analysis. For profile-line traces on (a) and (c), see Figures SI-4 and SI-5. (d) Digital close-up of (b) showing lattice fringes and inset oriented model cuboctahedron, enclosed by {111} faces and small caps of {100} and {110} type. Here the top/bottom void faces are clearly parallel to the lattice fringes.

The morphology of the rods is deemed unchanged during heat treatment, as the same crystallographic and morphological features are identified in Figure 2 comparing rods before and after heating. We note from this expanded study some important findings:

- Virtually every single rod examined after heat treatment shows a number of pores (light patches), much unlike cube samples discussed later.
- The diameters of pores are fluctuating much more narrowly than the size of rods, ranging from 1 to 12 nm with an average of 4–6 nm (as demonstrated in Figure SI-1). That means whatever the diameter of the rod, the pore diameter remains similar. For example, the voids found throughout Figures 2 and 3 range only from 2 to 11 nm across. The pore size is not proportional to the varying rod diameters and lengths but appears defined by the heat treatment.
- Most pores in Figure 2f (and even more so in Figure 3) are isotropic (e.g., compatible with regular octahedra). Their facets are aligned with the crystal lattices of the rod, as highlighted in Figure 3d.
- However, some of the newly heat-generated pores show elongated morphology along the rod direction, not reported previously. These voids in the shape of “negative rods” rather than “negative octahedral particles” could be an initiating stage of a later conversion of rods into hollow ceria nanotubes.^37,38^ While pore shapes vary, we find a trend that those pores, which show enclosure by straight facets, e.g., in Figures 2f and 3, have their straight border lines in projection along lattice fringes showing consistent faceted geometry, as further detailed in Figure SI-2.

**Figure 4:**
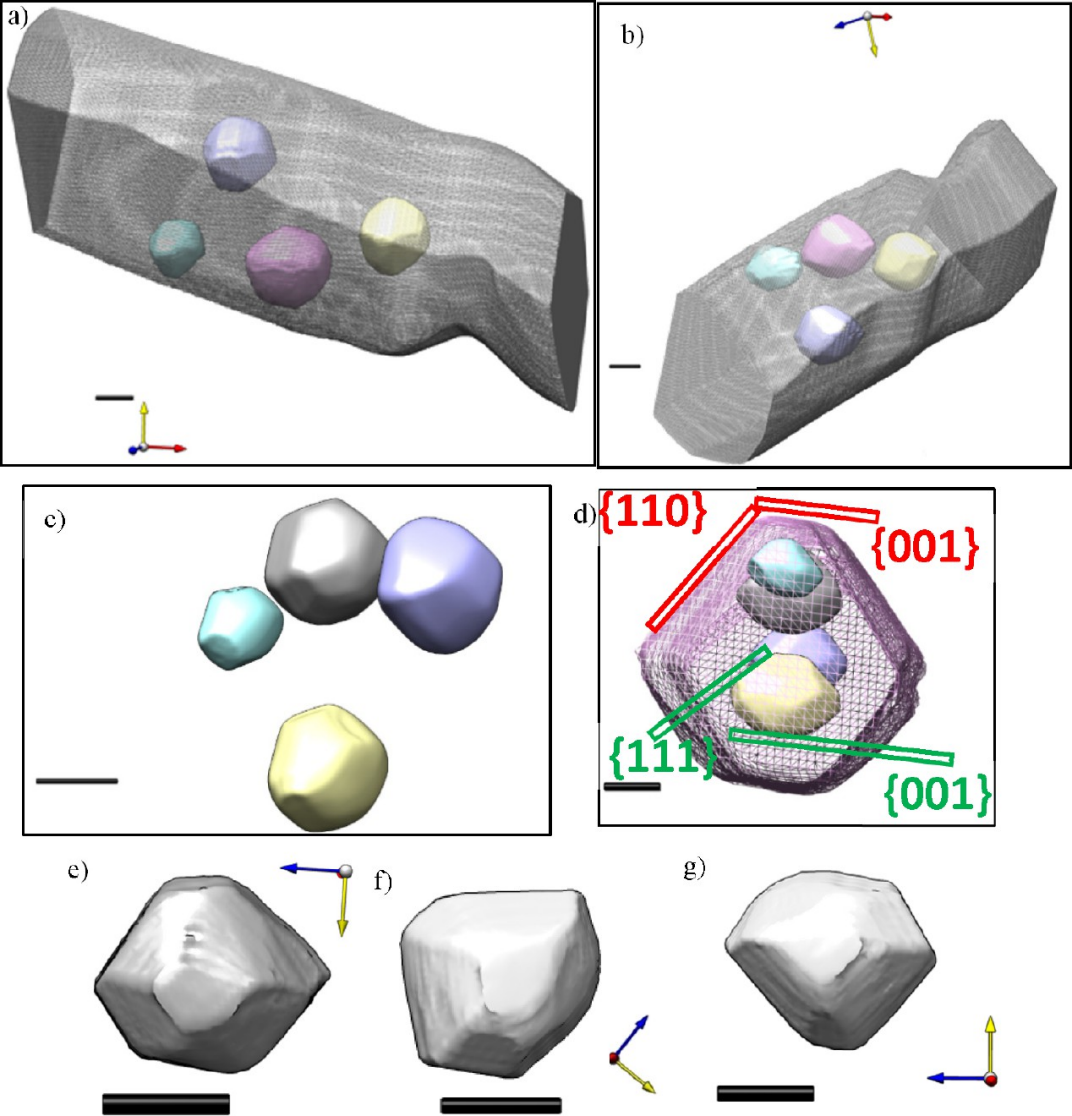
3D reconstruction of 800 °C-heated CNR segment containing four faceted voids. (a, b) Overall rod shape and relative internal void locations viewed perpendicular and along rod axis. (c) 3D reconstruction of the shape of individual pores with rod annulated. (d) Crystallographic index identification for rod (red) and pore (green). (e–g) A single pore in three viewing directions. All scale bars in (a–g) are 10 nm. Axes color code: *x* (red), *y* (yellow), and *z* (blue). The electron beam direction is indicated by the blue axis.

**Figure 5:**
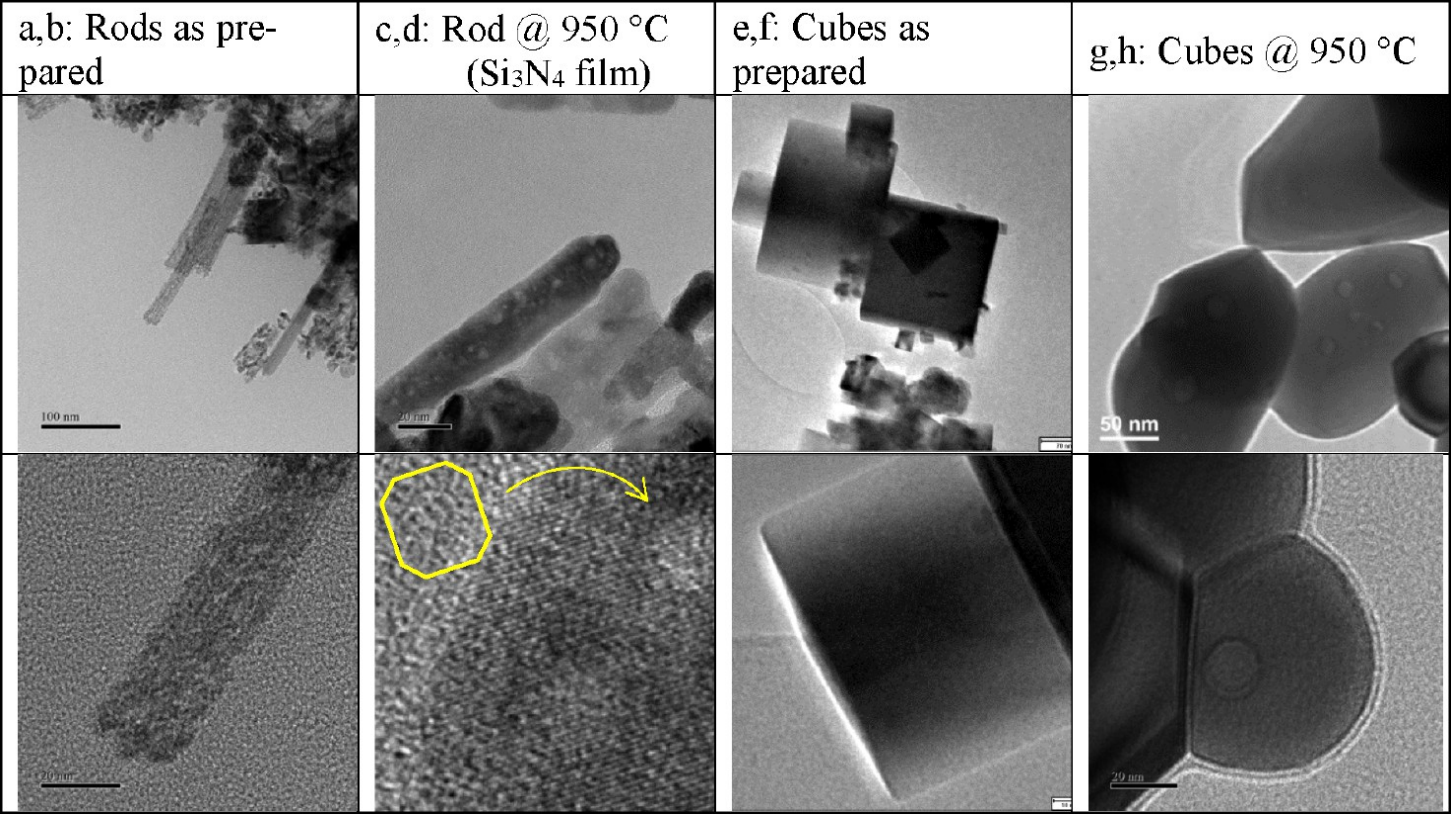
Occurrence of defects/micropores in rods before (a, b) and extended faceted mesopores in rods after HT heat treatment (c, d). Cuboctahedral model void as inset in (d). Absence of any defects or pores in cubes before (e, f) and occurrence of extended mesopores in cubes after HT heat treatment (g, h). Top/bottom represents two examples for each type of sample.

Figure 3 shows nanorods at various magnifications with a selection of pores along their length, suitable and chosen for tomographic analysis in Figures 4 and 5. For preparation details of the powders see previous work.^12^Figure 3a shows a segment of the rod 140 nm long suitable for 3D examination of both the external rod shape and the internal voids. Figure 3b shows a HRTEM image of the central part of the rods including four major pores suitable for tomographic reconstruction, with multiple sets of lattice fringes resolved, corresponding to (111) planes, as shown in Figure 3d. We can see that the fringes cross the void regions uninterrupted. This behavior suggests that the single crystalline structure is continuous in spite of the defects. Together with the absence of moiré patterns, this suggests that the voids occur within one single crystal grain, as of Figure 2f. Apart from slight variations of pore morphology (partially due to 2D-projection effects) we can see a consistency in the orientation of the features, with two opposite facets maintaining parallelism of exposed {111} planes, while the shorter sides remain parallel to the growth direction of the rod.

In Figure 3c another rod with average lateral size of 25 nm and pore sizes ranging from 4 to 7 nm is shown. This rod appears to have a higher concentration of pore-type defects per projected area than the one in Figure 3a,b, despite having similar diameters. This could be attributed to the pores in Figure 3a,b being larger, ranging from 7 to 11 nm.

### Three-Dimensional Imaging of Pore Distribution via Tomography

All three images in Figure 3a–c are the 0° central projections of tomographic acquisition series over ±60° angular range in 5° steps. A total of 13 2D images are underlying each acquisition series. Two series, at different magnification setting, have been recorded and are superimposed, as demonstrated in Figure SI-3. This helps benefiting from both high resolution and better alignment from a larger field of view. Because of various nonlinearities (strain and lattice contrast, see discussion below), standard computed tomography is not applicable for bright-field TEM at high resolution, without special mitigation of scattering contrast artifacts. While EFTEM and HAADF-STEM have been proposed for this situation,^17,39,40^ these require extra peripheral equipment and more complex image acquisition, not always available.

Heat treatment of ceria nanorods can result in strain around pores in single-crystalline nanorods, which contributes imaging artifacts, manifest as dark patches, through modified Bragg scattering. Electrons are locally removed from image intensity without the object being thick. These features are detrimental to electron tomography, as variations in gray values are interpreted as changes in thickness × density upon reconstruction. For this reason, ceria nanorods are good candidates for “geometric tomography” (GT) to reconstruct nanostructures based on binarized intensities. However, the standard condition for GT of objects being axially convex is not met here.

We are therefore exploring an innovative approach in which we at first subdivide the image information into multiple images, starting with the (solid) rod as a whole, followed by selected pores, with each pore being treated one after another. For each image subseries, the GT conditions are fulfilled. Following separate reconstruction, the resulting 3D models are superimposed as if they form one single reconstruction. The tomographic reconstructions obtained in this work are therefore innovative 3D models of the mesoporous nanorods and simultaneously reveal pore-shape information as well as the location of the pores with respect to the surfaces of the rod.

On the other hand, geometric tomography (GT)^34^ has already been demonstrated as a fast and low-complexity short-cut to use bright-field TEM for suitable nanoparticles.^33^ GT is also termed the “shape-from-silhouette” technique. However, the main applicability condition for GT is that all objects are shaped in “axially convex” cross sections. That means each 2D section perpendicular to the rotation axis must have convex shape, as otherwise any concavities would be filled up and enlarged into a geometric body called the “convex hull”. For the external nanorod shape we expect axial convexity to be fulfilled. The same applies for each individual void in isolation, if contrast is inverted into a “positive particle”. However, the combined object (rod with multiple overlapping voids) would classify as mixed convex–concave. Here we propose a new strategy, modified from Saghi et al.:^33^ First, we separate the rod-image binarization from the void-image binarization. Then we apply back-projection two times: first to reconstruct the rod shape only as if there are no voids and second to reconstruct the voids one by one (in their original 3D space position relative to each other) as if there was no finite rod around them. The resulting 3D models of rod and voids are then intercalated into a combined 3D reconstruction.

The lower magnification images like Figure 3a allowed us to reconstruct a long enough representative segment of the rod, while the higher detail in images like Figure 3b was used to extract the faceted geometry of the voids. The final reconstructed volume of interest comprises the outer morphology of the rod along with four internal features of interest. The 10 nm scale bar can be used to verify the lateral dimensions of the rod, which is 35 nm wide by 45 nm tall with defined facets along the (220) and (100) planes. The distance between the diagonal faces is 40 nm.

We present the reconstruction of the rod surface as a transparent mesh while the pores are inserted as solid surface rendered “particles”, viewed either perpendicular (Figure 4a) or along the rod axis (Figure 4b). The location of the pores within the rod is accurate, as the locations of pores and rods were not altered relative to the 3D data space during the separately executed back-projections. Both views confirm the existence of a preference orientation of the pores with respect to the faceted sides of the rod and the rod axis: The smaller flat surfaces at the top and bottom of each pore are parallel to the top and bottom surfaces of the rod. In a similar way, the longer diagonal sides of the pores are parallel to the diagonal sides of the rod. The missing wedge artifact deforms the sides of each feature. In the view shown in Figure 4c, the pores appear to end in flat surfaces at the top, bottom, front, and back, while the sides terminate in a point. It is expected that these sides terminate in (100) surfaces like the others. This orientation is further confirmed by postprocessing in Figures SI5 and SI6. This orientation relationship emphasizes the single-crystal nature of the rod and equivalence in shape equilibration for each facet pore, similar to how octahedral nanoparticles would adopt a common relationship if grown on a connecting single-crystal substrate by epitaxy. The void shape also matches the shape of an “oriented attachment” building block, which plays a role during hydrothermal growth, as imaged in Figure SI-2.

When we remove the rod from view (Figure 4c), we can appreciate the consistency in shape and orientation of the pores, which present flat surfaces at the top and bottom. We can also see that the larger pores, such as the ones at the top and right in Figure 4c, have more defined geometries than the smaller pore at the far left of the image. Finally, the bottom row, Figure 4 e–g, shows the reconstruction of a single pore in isolation.

The overall crystallography is indexed for rod and pores in Figure 4d: The geometry is symmetrical and is composed of smooth surfaces. For reasons of single crystal geometry the rods and pores must have differing facet distributions and therefore differing angles relative to the flat {100} type reference planes which are in common. The diagonal {110} planes for the rod are well distinguished in the reconstruction from the less steep {111} facets of the cuboctahedral pores.

The presence of pores inside the body of the rod, which do not reach the surfaces, requires careful definition of functional parameters in prospective applications, such as the surface area/volume ratio (see also the Discussion section). The conventional ratio (*S*_ext_/*V*), using only external surfaces, remains nearly the same after heat treatment due to the diameter and volume not changing. However, if we introduce a total *S*/*V* ratio via *S*_tot_/*V* = (*S*_ext_ + *S*_int_)/*V*, the formation of surface significantly increases due to internal surface, e.g., for the porous rod shown in Figure 4 by ∼36% compared to its nonporous counterpart. The applicability of *S*_tot_ instead of *S*_ext_ for predicting functional performance depends on the gas permeation of the very thin separation walls.

### Cross-Sectional Analysis of Ceria Nanorods

A main advantage of tomography is that the resulting 3D models can be sliced into cross-sectional planes. At first, we analyze intensity profiles evaluated from Figure 3a, displayed as Figure SI-4. The residual contrast inside the pores relative to the carbon background clearly confirms that the light patches are never through pores or holes (as of Figure 1f) but must be internal voids as proven by the tomography in Figure 4. Next, we construct three cross-sectional tomographic reconstructions from the tilt series behind Figure 3c, shown in Figure SI-5a–c. The reconstructed cross sections show in white the outer morphology of the rod. This consists of a six-sided polyhedron of 2-fold rotational symmetry with prominent {110} facets cut by shorter {100} facets, with occasional {110} capped edges. These intersections are credited to host most catalytically active sites.^7^ The shape is also compatible with previous 3D models for ceria nanorods.^10^ The tomography results also show the pores as symmetrical with defined facets consistent with mainly {100}-truncated {111}-octahedra. The geometry appears to align with the edges of the rod, coinciding in their shorter {100} sides and their more prominent {111} facets. As for the location of the pores, the reconstructions show them to be fully enclosed by the rod. They seem to appear at random lateral locations within its volume. In Figure 4b, the distance between pore and surface is never smaller than 1.4 nm.

Furthermore, it is important to verify the isotropy and deviations from it: In contrast with other forms of tomography, electron tomography does not allow a full axial rotation of the sample. This is limited by the goniometer and the projected thickness of the specimen at high tilt angles, which impedes transmission imaging. The missing information results in a “missing wedge” artifact. To distinguish the effect of the missing wedge from the morphology of the sample, we include a theoretical reconstruction of a perfect cylinder simulating the same imaging conditions as our experimental results (Figure SI-6). We can see that the top and bottom of the reconstruction present the expected circular cross section, while the area of missing information appears elongated and distorted. However, the missing wedge would not introduce any extra capping of the rod or void shapes on the top and bottom ({100} facets). There is no facet detected in our work, which would not be visible in at least one 2D image, although the missing wedge might lengthen {111} type facets.

To study the pore–rod relationship in more detail and to judge possible influences of incomplete data acquisition (120° instead of 180° tilt series), we apply another modified GT algorithm: In Saghi et al.,^33^ the standard GT algorithm was contrasted with an even further data-reduced concept, labeled “shape-from-contour”: rather than binarizing object projections into regions inside vs outside the radiation shadow, each object surface line in each image is “edge-enhanced” into a bright line while the areas both inside and outside the objects are blackened. We show here for the first time that this algorithm can successfully deal with porous objects, such as a pore inside a rod, by using the cross section (i) arrowed in Figure 3c. The selected cross section appears as a 1D line on each image in the tilt series, with only four pixels retained: the four transition positions from rod/vacuum and rod/pore.

The advantage of the shape-from-contour method relative to the earlier GT algorithms lies (apart from data compression) in its ability to (at least partially) simultaneously process binary objects fully enclosed inside larger objects, as the thin back-projected lines are less likely to override inner contour details, which would be completely wiped clear by the shape-from-silhouette approach. Figure SI-7c is an encouraging result, as it can be safely interpreted for both rod shape and pore shape from a single cross section and thereby rendering the applied segmentation optional. We plan to expand on this reasoning in a forthcoming publication.

### Influence of Heat Treatment on Ceria Nanorods

Figure 5 shows a selection of high-resolution TEM images comparing samples of nanorods and nanocubes as-synthesized and after high-temperature (HT) near-sintering heat treatment, complementing earlier work in refs (12 and 13). In brief, we observe rounding and shortening of the nanorods and even more pronounced loss of edges and corners in the cube morphology. We note the prevalence of a few sharp corners after heat treatment, e.g., cubes at 950 °C (a). By comparison of the as-prepared samples, the monocrystallinity of the rods is more pronounced. This is consistent with the similar comparison shown in Figure 2. Such a TEM on furnace-heated samples is typically performed by heating the powder in a suitable container and then drop some grains afterward onto a (cool) support film/grid. To evaluate the possibility of heating the powders on the TEM grid, we trialled an amorphous Si_3_N_4_ film. The upper temperature is a challenge due to brittleness and possible reaction in air. In Figure SI-8 we show that Si_3_N_4_ is safe to be utilized at 950 °C.

As expected, pores have formed in the rod morphology upon heat treatment. The size of these pores is similar to the ones found after heating at lower temperature.^13^ The shape of the pores does not appear to have lost the facets analyzed in the previous sections, regardless of the rounding of the rods. The pores do not appear to be closer to the edges of the rod than in Figures 2 and 3, which suggests that they have remained inside the structure. The cube-shaped nanoparticles^12,45^ also developed pores upon heat treatment to this higher temperature, which is a new finding. They are not as common as in the rods with only few pores per cube. However, they tend to be larger in size than the pores found in the rods.

### Molecular Modeling of Voids in Ceria Nanorods

#### Classical Molecular Dynamics

To complement our experiments and help rationalize the structure, shape, and evolution of voids within ceria nanorods, we performed a combination of classical molecular dynamics (MD) and density functional (DFT) simulations.

**Figure 6:**
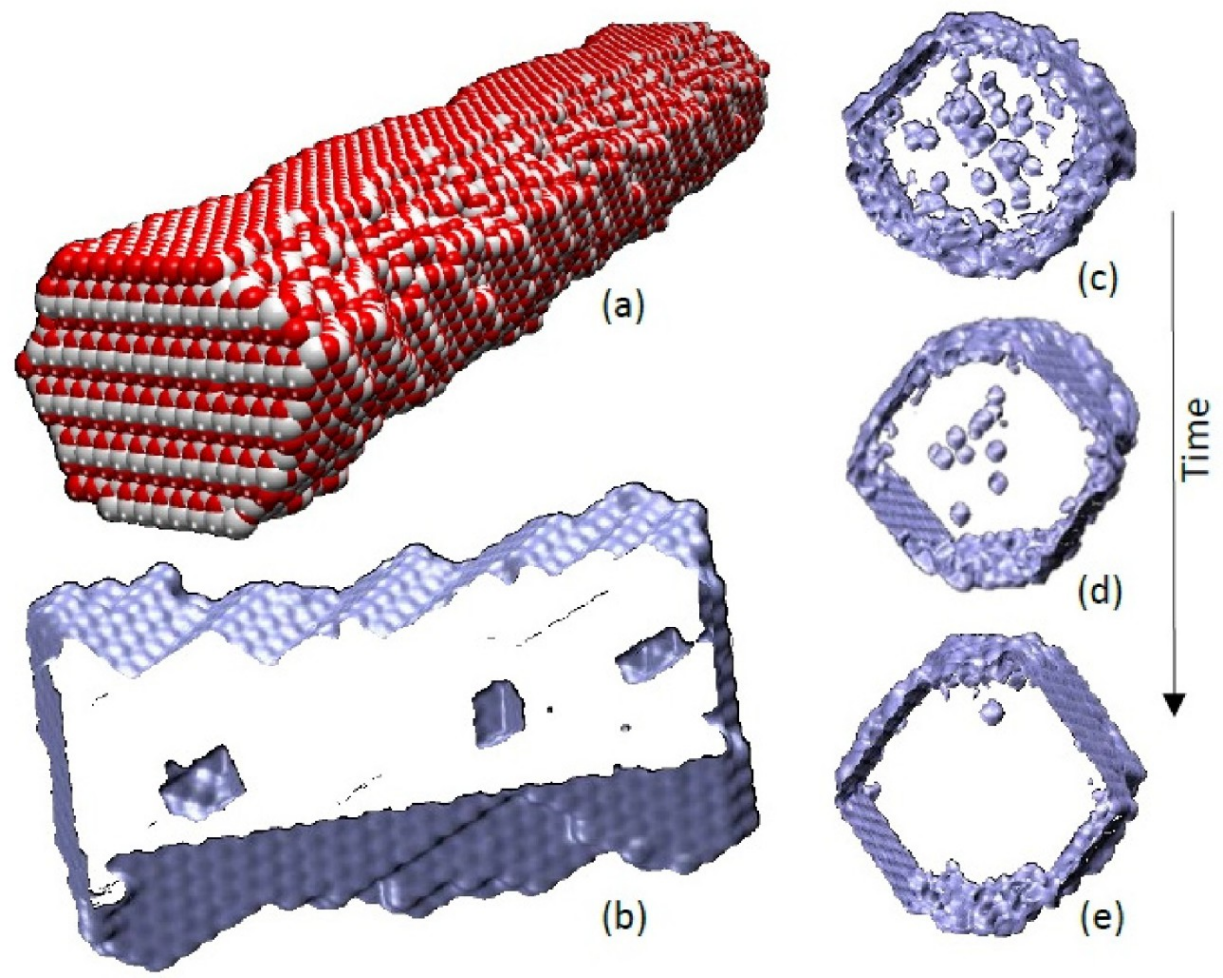
Molecular modeling of porous ceria nanorods. (a) Structure of a ceria nanorod with [211] growth direction; oxygen atoms are colored red, and cerium are white. (b) Surface rendered model of a slice cut through the nanorod in (a) showing voids that have evolved via the agglomeration of cerium and oxygen vacancies within the nanorod. Single vacancies and vacancy clusters are also present but are not shown to reveal more clearly the structures of the (larger) voids. (c–e) Snapshots of an MD trajectory depicting a segment cut through a model ceria nanorod with [110] growth direction revealing the gradual annihilation of voids and vacancy clusters during simulated annealing. The diameter of the model nanorod is about 4 nm.

A model CeO_2_ nanorod, with [211] growth direction (Figure 6a), was heated under molecular dynamics simulation to within 90% of its melting point. Analysis of the nanorod revealed that surface Ce and O vacancies spontaneously evolved as surface atoms moved off their lattice positions. Occasionally, the vacancies would migrate toward the center of the nanorod. We hypothesize that voids in ceria nanorods, observed experimentally, evolve from the agglomeration of these vacancies in the body of the nanorod. To test this hypothesis, we introduced 4500 vacancies (1500 cerium and 3000 oxygen), at random positions, into a (15972-atom unit cell) nanorod with [211] growth direction. The nanorod was then heated, under MD simulation, to within 90% of its melting temperature. Analysis of the atom trajectories revealed that both oxygen and cerium vacancies were mobile within the nanorod and agglomerated to form voids. In particular, a segment, cut through the nanorod during the MD simulation (Figure 6b), shows three voids that have evolved from the agglomeration of vacancies.

**Figure 7:**
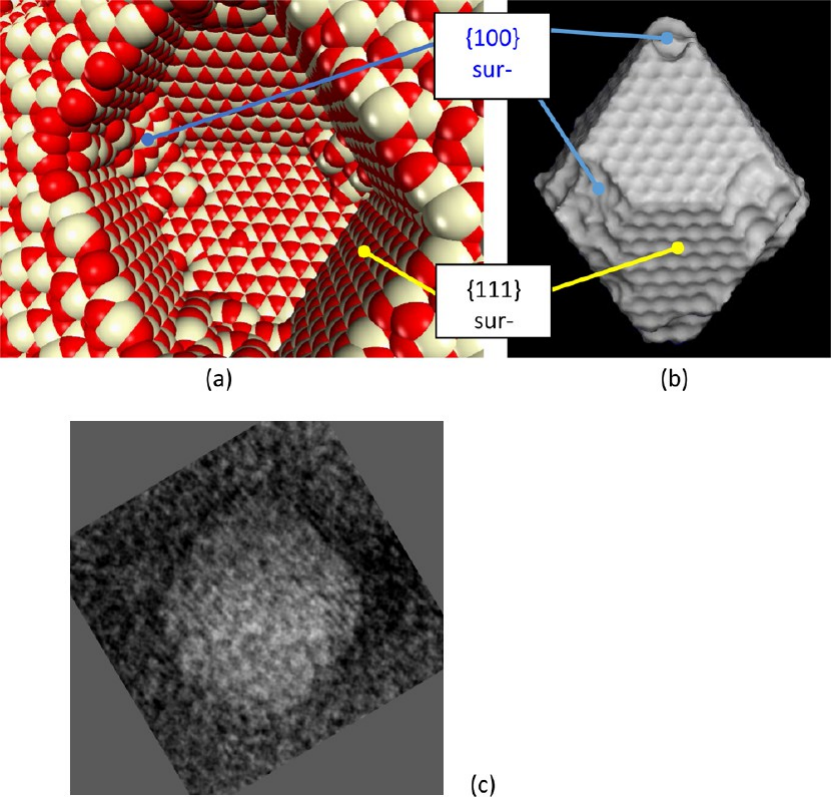
Atom level (model) structure of a void within crystalline ceria. (a) Sphere model representation of the atoms; cerium is colored white, and oxygen is red. (b) Surface rendered model of the void revealing more clearly the truncated polyhedral morphology, to be compared with Figure 4d. (c) Experimental faceted void with lattice fringes reoriented to match orientation of (b). The diameter of the (model) void (a, b) is ∼4 nm.

The voids within the nanorods (Figure 6b) appear to have octahedral morphologies, but the voids are small, which makes morphological characterization difficult. Accordingly, we generated a model of a much larger void. In particular, a spherical void was generated at the center of a simulation cell of crystalline ceria by removing a stoichiometric number of Ce and O atoms. The system was then heated, under MD simulation, to within 90% of its melting point. During the simulation, the spherical void transformed into a truncated polyhedron with eight {111} surfaces truncated by six {100} surfaces (Figure 7), in accord with our experimental findings.

The surface view of the model (Figure 7b) is suitable for direct comparison to the projected appearance of experimental voids, as follows: In Figure 7c, the top right facet of (111} type matches the equivalent top right facet in Figure 7b, while the top corner in both Figure 7b and Figure 7c is capped, indicating the {100} occurrence. For a 3D comparison of model and experiment we point to Figure 4d, which shows tomographic reconstructions oriented with {111} and {100} suitable for comparison with Figure 7b. Overall, the “negative particle” concept is confirmed, while also acknowledging the occasional asymmetry of octahedral facets and cubelike caps as found in Figure SI-2, leading to distorted octahedra.

#### Density Functional Theory (DFT)

**Figure 8:**
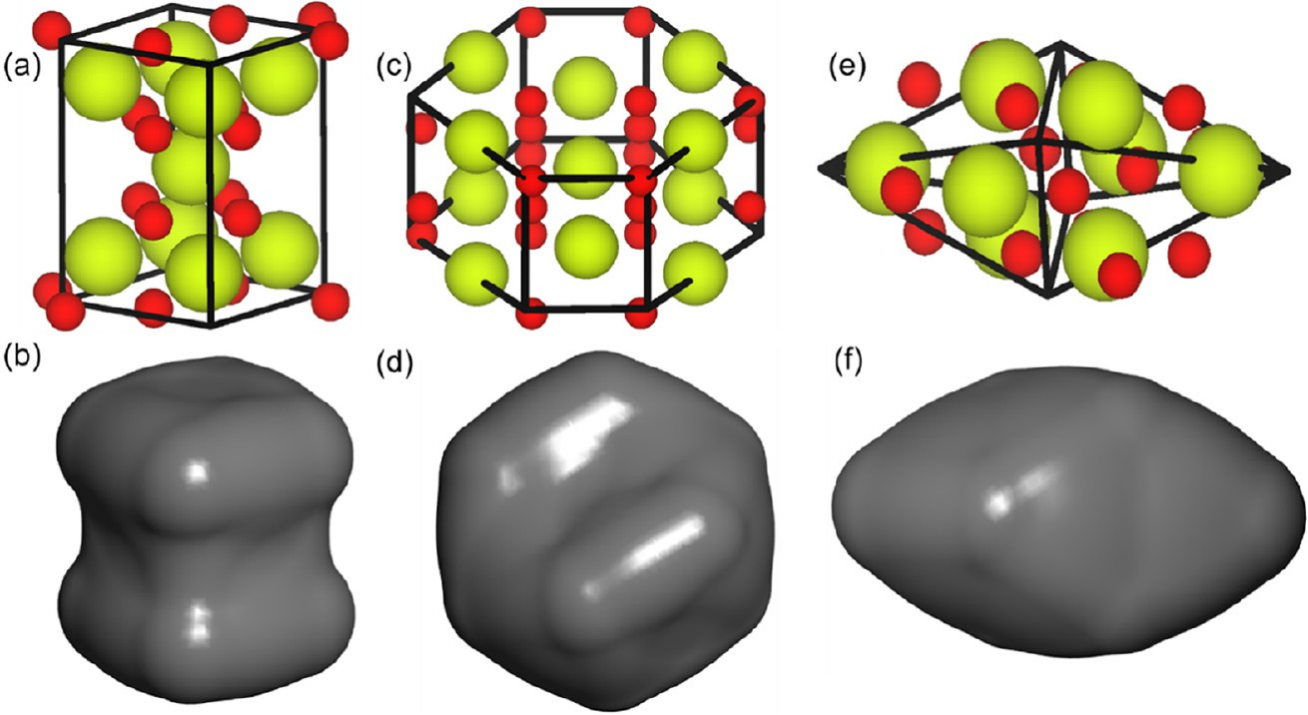
Voids of different shapes have been introduced in a cube comprising 256 CeO_2_ units: (a, b) cube-like, (c, d) hexagon-like, and (e, f) octahedron-like shaped voids.

DFT calculations of a supercell comprising 256 CeO_2_ units were performed to determine whether there is a difference in energetics of the voids with respect to shape. A small number of stoichiometric CeO_2_ units were removed to form cube-like, octahedron-like, and hexagon-like shapes as shown in Figure 8. It is clear that the stability of the voids is related from the thermodynamic point of view to the surfaces that are expressed in the cavity. Cube-like voids are the least stable because they express {100} surfaces with an energy of 3.14 J/m^2^. Octahedron-like voids are more stable as they express {111} surfaces (2.48 J/m^2^). The hexagonal prismatic-like voids express both {111} and {110} surfaces and lie between the cube-like and the octahedron-like voids in terms of energy (2.68 J/m^2^).

#### Computational Prediction of Catalytic Activity

Nanoceria can capture, store, and release oxygen from its surfaces. This ability, named oxygen storage capacity (OSC),^41^ enables oxidation/reduction reactions that are central to many applications. For example, environmental “clean-air” technologies use nanoceria to release oxygen and catalyze CO to CO_2_ while simultaneously (catalytically) reducing harmful NOx emissions by capturing oxygen.^2^ Nanoceria can also act as an enzyme mimetic or “nanozyme” by capturing and releasing oxygen from its surfaces to modulate concentrations of oxygen species in cellular environments.^42^ It is therefore important to understand how voids within nanoceria might influence its ability to release oxygen from its surface in catalytic reactions.

**Figure 9:**
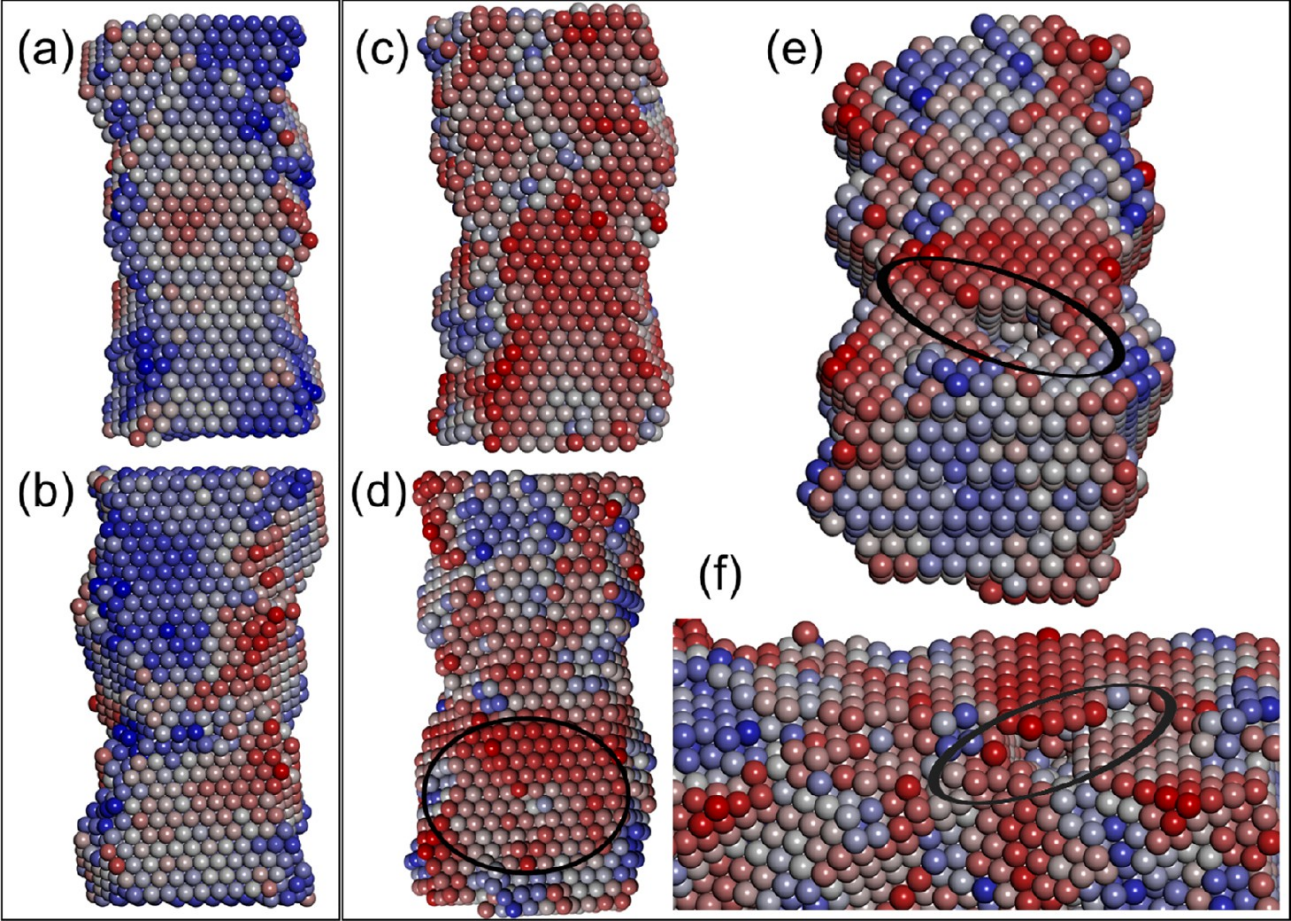
Activity maps of a ceria nanorod, with [211] growth direction, with and without voids. Only oxygen atoms are displayed and colored according to their Madelung energy by using a red–white–blue sliding scale where red indicates oxygen that is energetically easy to extract and blue indicates oxygen atoms that are difficult to extract. The nanorod is composed of two large {111} surfaces shown in (a) and (b) for the nanorod without voids and (c) and (d) for the nanorod containing voids. (e) and (f) are views of a void close to the surface shown in (d); atoms were removed to show the void, and ovals indicate the position of the subsurface void.

The energy required to extract oxygen from the surface of nanoceria “is a simple yet powerful activity descriptor”.^43^ Reactivity maps, where oxygen atoms are colored according to the energy required to extract them from the surface (calculated via Madelung energies^44^), are shown in Figure 9. The oxygen atoms are colored on a red–white–blue sliding scale, where red represents oxygen atoms that require the least energy to extract and oxygen atoms colored blue require the most energy to extract.

Calculated reactivity maps of the nanorod with and without voids (Figures 9a,b and 9c,d, respectively) show very different surface patterns. In particular, images reveal that it is (thermodynamically) easier to extract surface oxygen from a nanorod with voids (oxygen atoms predominantly colored red) compared to ceria nanorods without voids (surface oxygen atoms predominantly colored blue). Accordingly, the simulations predict that voids activate {111} surfaces.

## Discussion

Following the tomographic reconstructions, the “shaped-void” defects in this study can now be clearly categorized with respected to speculative alternatives, as sketched in Figure 1. We can safely exclude for our materials the two cases of through holes and surface pits (Figure 1e,f) as all voids are internal. The atomic-number contrast between rod and void is also far too large for oxygen-only vacancy-rich regions. That leaves option Figure 1d, internal faceted voids, as the only compatible one. Option Figure 1c, a high concentration of subnanometer small defects, is continued to be believed as the likely precursor situation before heat treatment, essential to accumulate the high internal energy which is driving consolidation into fewer but larger and shape-equilibrated defects. The actually identified preferred *octahedral* shape for the negative-particle voids is consistent with both molecular dynamics simulations and DFT calculations, confirming its lowest energy status and a dynamic pathway to its formation.

The existence of such unfilled space in this study is to be seen with respect to volume loss upon the NaOH triggered conversion of Ce(NO)_3_·6H_2_O to CeO_2_ with Ce(OH)_3_ as an intermediate solid phase. However, our results show that any precursor hydroxide phase expected to exist during the hydrothermal synthesis does not act via template synthesis to shape future voids. There is no phase separation followed by dissolution of the more volatile phase (as e.g. in dealloying of AuAg nanoalloys or in the Vycor process of glass-in-glass phase separation^31^). The void shape is rather an equilibration of surface energy after the (pure) CeO_2_ composition is achieved, with the posthydrothermal heat treatment supplying the mobility to both cations and anions.

In our atomic modeling calculations, vacancies of O and Ce, preloaded into a rod model, are found in their majority not to persist through heat treatment, instead escaping through nearby surfaces. It is therefore essential that pre-existing internal microvoids (causing the rough contrast in Figure 5b, also reported by Florea et al.^10^) are assumed, which grow by ripening without escaping the rods. However, it is also found that formation of negative-particle shape voids can happen spontaneously, well distant from the surface, and therefore does not require phase separation and chemical impurities, or a sacrificial spacer phase, if the right conditions are applied, starting from a pure material enriched in point defects. Modeling confirms that the shape assumed by the voids is determined by local equilibrium of surface facets and not via template casting of any earlier phase.

The applied heat-treatment temperature of 800 °C is “medium”, in the sense that it is below the known sintering-onset *T*_sint_ (expected around 900 °C) for ceria nanostructures but above routine annealing applied to such nanomaterials (<600 °C). Our comparative high-*T* experiments at 950 °C thus reveal the differing behavior of external shape and internal voids: external surfaces start to flow under the influence of surface tension, assuming rounding, while the internal voids remain trapped inside the rods and do undergo less rounding or growth.

Interestingly, cube samples, which so far have never been reported to grow negative-particle voids, due to their isotropic growth, have also been found with at least some voids (much less than for rods), surviving the 950 °C external rounding of the cubes.

Images such as Figure 2f indicate that some voids are rather elongated, of “negative-rod” shape rather than “negative-octahedron” shape. The long axis of those is always aligned with the rod axis, and they tend to prefer locations central to the rod. It is therefore easy to extrapolate that further (longer holding time) heat treatment might trigger growth and merger of these rod voids, which would end up forming ceria nanotubes, like those reported in refs (37 and 38).

Some secondary observations referring to strain are of interest as well, although these had to be suppressed to enable tomographic reconstructions: Many voids in Figure 3 are enclosed in dark patches, representing modified Bragg scattering in those bright-field images. This must be residual strain after formation of the voids from earlier microdefects, not annealed out at the medium temperatures used. The effect is maximized near sharp corners enclosing the voids. Apart from those strain effects, the lattice is however of greater crystalline quality compared to before any heat treatment (Figure 5a), indicating the absence of microdefects. This strained neighborhood around pores is also found persisting in 950 °C heated TEM samples. The bright-field images of the rods (Figure 3) also show a dark stripe of constant width running along the longitude of the rod. This is consistent with the pseudohexagonal cross section of the rod and underpins the flat central surface part found via tomography (Figure 4b). However, this is pure mass–thickness contrast differentiated from the aforementioned dark patches encircling the defects, which are strain contrast.

Apart from inducing pores, it is well-known that heating can induce transformation of less stable CeO_2_ surface facets (e.g., (110)) via subfacetting and appear in HRTEM as a zigzag edge in projection (see refs (45 and 46)). While this phenomenon can be traced in our modeling (see Figure SI-9), the rods selected in the present work have flat external surfaces. No subfacetting occurs in the modeling of the voids, while we occasionally see flattened edges at cuboctahedra of {110} type on micrographs of the ⟨110⟩ viewing direction.

The location of these cavities inside the body of the nanorods raises interesting questions as to the performance in applications, as porosity in similar (but more open) ceria nanostructures has been found beneficial.^14^ Here, our simulations predict that it is easier to extract oxygen from the surfaces of ceria nanorods, which contain voids, compared to nanorods without voids. Reduced energetics for oxygen extraction, to create vacancies, impacts upon the catalytic activity, oxygen storage capacity, and (vacancy-driven) oxygen transport within ceria.^2,43,47^

## Conclusions

Hydrothermally synthesized ceria nanorods heat-treated at 800 °C resulted in development of internal voids of well-faceted “negative particle” shape, alongside higher crystalline quality with lower point defects, while external rod morphology remains unchanged. For the first time, although at lower concentration, cavities are also found in ceria nanocubes. However, heating to 950 °C results in surface rounding, onset of sintering of neighboring particles, while the internal cavities still persist.

A modified geometric tomography methodology, adapted to the case of multiple pores inside a convex rod shaped sample, is presented to successfully reconstruct the pore shapes and rod cross sections by using bright-field TEM only. The cuboctahedral shape is confirmed for most voids, with some of them forming elongated negative rods. None of the reconstructed voids or cavities have connection to the surface. However, the separation wall of around 1.5 nm would be well permeable for atomic oxygen diffusion and would render the internal voids possibly contributing to catalytic activity.

Computational modeling has further elucidated the evidence from the experiments: first, molecular dynamics revealed that cavity formation from aggregation of point defects can be a spontaneous process without the need of a spacer phase to enforce cavities. Second, density functional theory confirms that the preferred void shape is octahedral (negative-particle) by using surface energy balance similar to predicting positive particle shapes. And finally, calculations of multineighbor oxygen coordination energies from rods with and without cavities confirm that surface oxygen feels the presence of subsurface cavities and benefits from lower extraction energy.

It therefore appears that carefully chosen intermediate temperature for heat treatment of known active shapes of nanoceria, e.g., rods and cubes, can be beneficial to further boost catalytic performance in those materials.

## Supporting Information Available

Statistical analysis of pore diameters; pore geometries relative to the nanorods; 3D reconstructions using modified geometric tomography methods and of a perfect sphere; influence of TEM sample support; an extensive description of the rationale for modeling (PDF)
Video S1: dynamics of atom motion on the modeled nanorod surface (MP4)
