# Supplementary material for: Tomographic Study of Mesopore Formation in Ceria Nanorods
Source: J Phys Chem C Nanomater Interfaces. 2021 May 3;125(18):10077–89. doi: 10.1021/acs.jpcc.1c01221 (PMC8279707; doi:10.1021/acs.jpcc.1c01221)
Supplement: Supplementary file 1 — jp1c01221_si_002.pdf [file jp1c01221_si_002.pdf]

Tomographic Study of Meso-pore Formation in Ceria Nanorods

Supporting Information

C. Brambila[1], D. C. Sayle[2], M. Molinari[3], J. Nutter[1,5], J.M. Flitcroft[3], T. X. T. Sayle[2], T. Sakthivel[4], S. Seal[4], G. Möbus[1]

[1]Department of Materials Science and Engineering, University of Sheffield, Sheffield S1 3JD, U.K.
[2]School of Physical Sciences, University of Kent, Kent CT2 7NZ, U.K.
[3]Department of Chemistry, University of Huddersfield, Huddersfield HD1 3DH, U.K.
[4] Advanced Materials Processing and Analysis Center, Nanoscience and Technology Center (NSTC), Materials Science and Engineering, (MSE), College of Medicine, Biionix Cluster, University of Central Florida, Orlando, Florida 32816, United States
[5]The Henry Royce Institute, Sir Robert Hadfield Building, Sheffield S1 3JD, U.K.

Main manuscript: ACS, Journal of Physical Chemistry C, doi.org/10.1021/acs.jpcc.1c01221

In this supplementary information, we report further details concerning background information behind the figures in the main manuscript.

(i) A statistical pore size analysis to put into context the chosen pores for tomographic analysis, which are confirmed as of "typical" size (Fig SI-1)

(ii) A further micrograph confirming the well-facetted nature of pores and their alignment to the main rod axes (Fig SI-2), as well as the formal relationship of pores and building blocks within Oriented Attachment growth.

(iii) Examples from the tomographic tilt series used for Fig 4 of the main manuscript. (Fig SI-3)

(iv) Profile analysis, geometric tomography, and shape-from contour methods applied to ceria nanorods. These results confirm the findings reported throughout the paper. The reconstructions in Figs SI-5 – SI-7 are also meant to present the advantage of using binarized images or even contour methods for efficiency.

(v) Example images about TEM sample support, Fig SI-8, carbon vs Si-nitride.

(vi) An extensive *Rationale* behind the simulations presented in the main manuscript. (Figs SI-9 to Si-12)

(vii) A video file of the dynamics of atom motion on the modelled nanorod surface is provided (outside this word-document), to accompany Figs SI-9 to SI-12. The video file is uploaded alongside the supporting information.

Supplementary Experimental Figures:

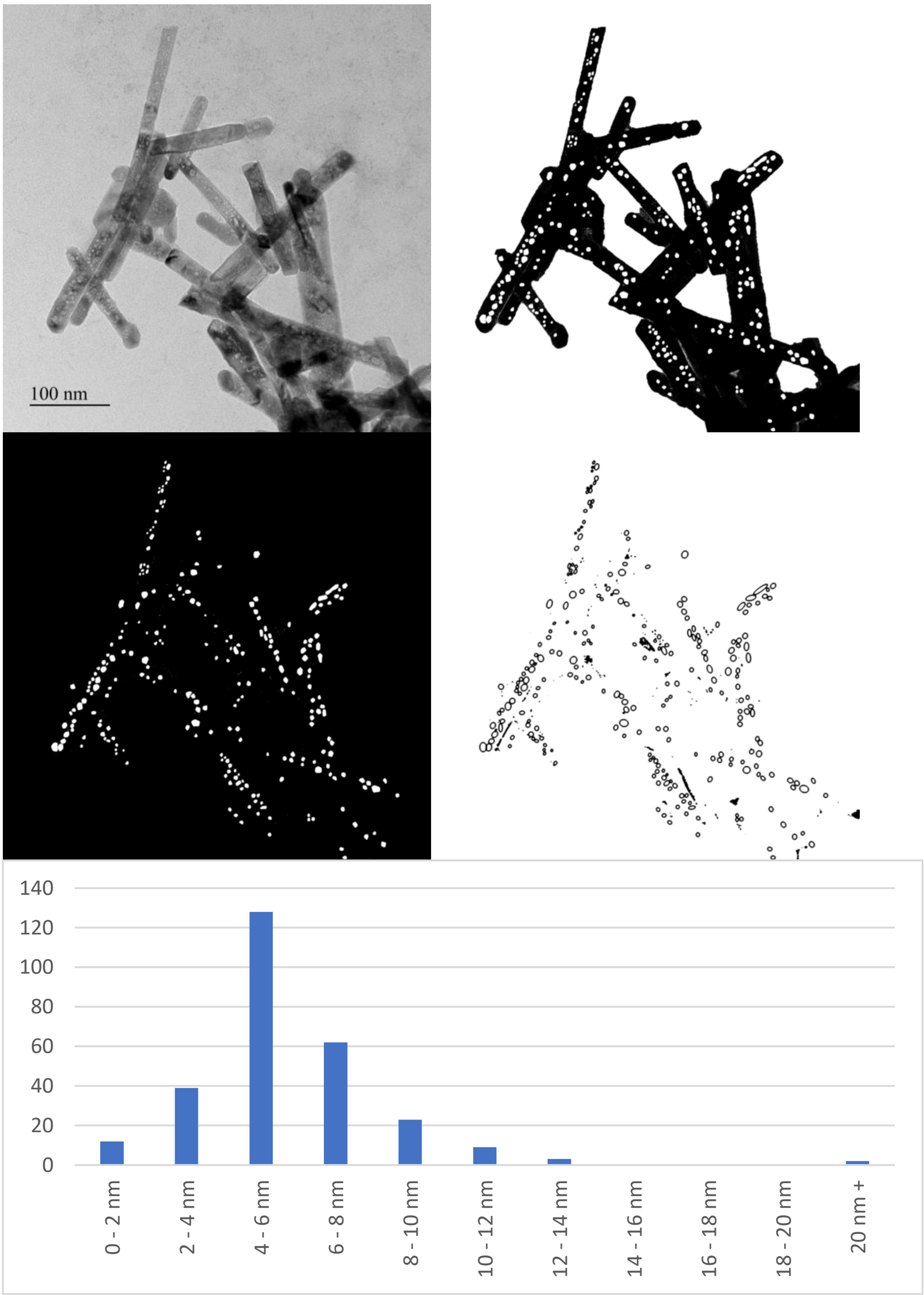

Figure SI-1. TEM-based pore size analysis using ca. 250 data.
Contrast Binarization, Pore detection, and Edge detection. Histogram of size distribution.

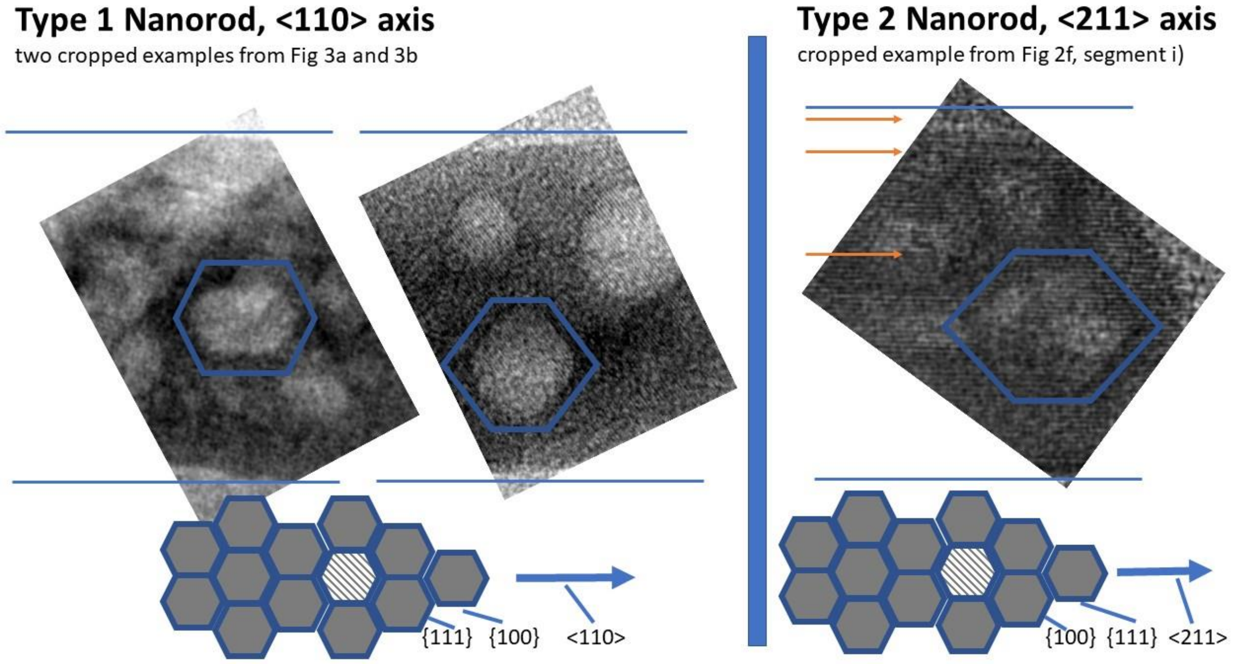

Fig. SI-2. Orientation of rods and voids for examples of rods from Fig 2 and 3 (main manuscript),
with rods re-oriented to have horizontal axes:
Main images: Selected voids with {111}/{100} facets, for Type 1 (<110> rod) and Type 2 (<211> rod).
Model images: Schematic of Oriented Attachment growth of Type 1 and Type 2 rods
(after Du et al, 2007, reference 16 of main manuscript):
Each of our facetted voids can be (formally) exchanged for one "building block" of the OA-growth mechanism.
Not to scale (regular hexagons used for simplicity).
Orange arrows indicate parallelity of rod faces (horizontal blue lines), lattice fringes and void facet direction.

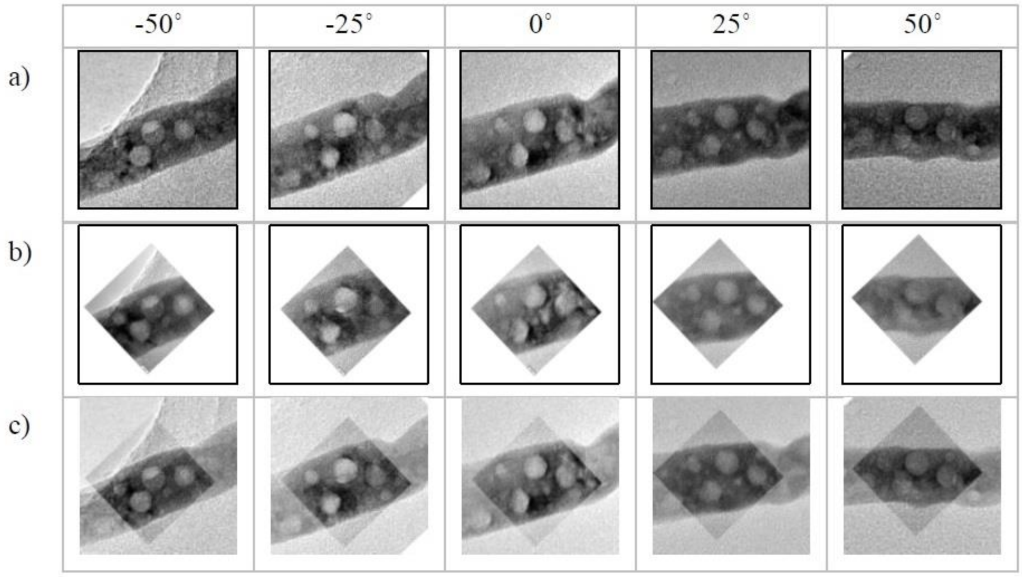

Fig. SI-3. TEM images representing samples from the tilt series over 120° for 3D reconstruction acquired with large field of view (a), for better alignment, and small field of view (b), for better resolution, and their superposition in (c).

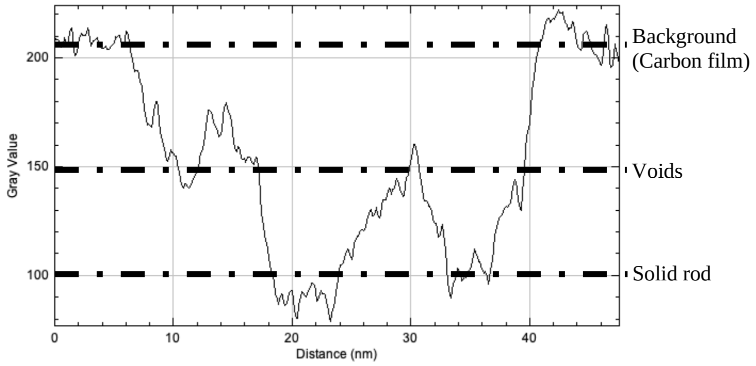

Figure SI-4. Profile of image intensity across Fig 3a of the main manuscript to highlight pore-vs-background contrast. The variation confirms that the voids are not through-holes.

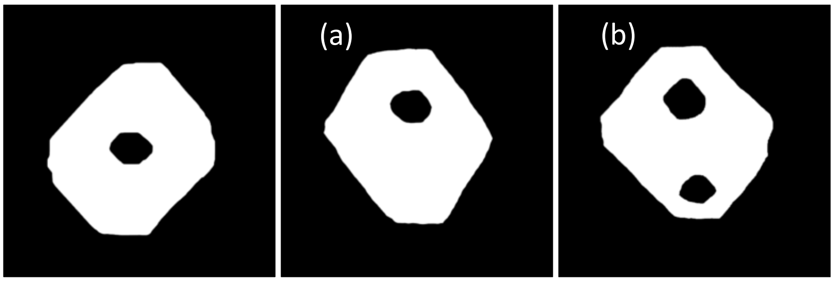

Figure SI-5. (a,b,c) Cross-sectional views of 3D reconstructions from Fig 3c of the main manuscript (located near profiles i,ii,iii), generated using Back Projection via Shape-from-Silhouette method.

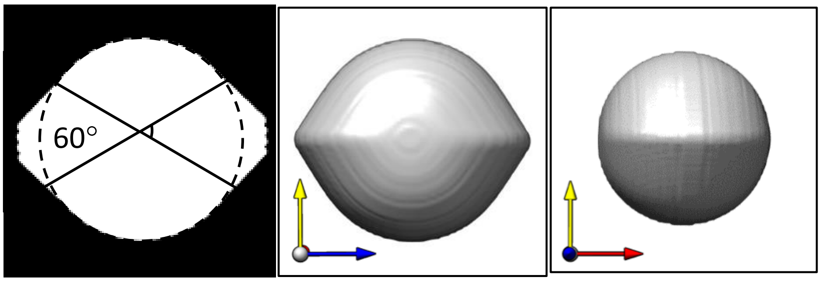

Figure SI-6. Visualisations of back-projected reconstruction of a perfect sphere to highlight the effect of the missing wedge.
Axes colour code: x (red, the rotation axis); y (yellow); z (blue)

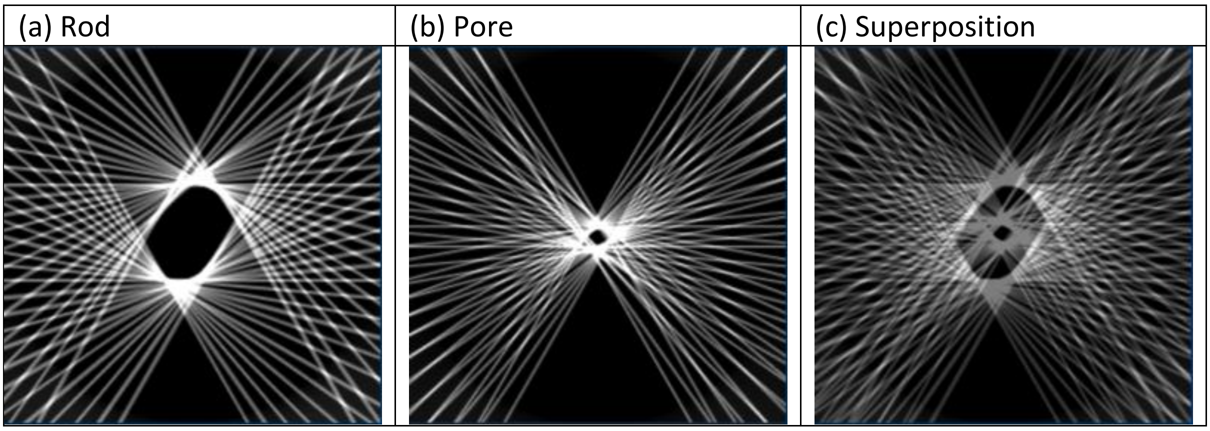

Figure SI-7. Cross-sectional views of 3D reconstructions from Fig 3c of the main manuscript, profile (i), generated using Back Projection via Shape-from-Contour method.
The reconstructions of rod (a) and pore (b) are superimposed in (c).

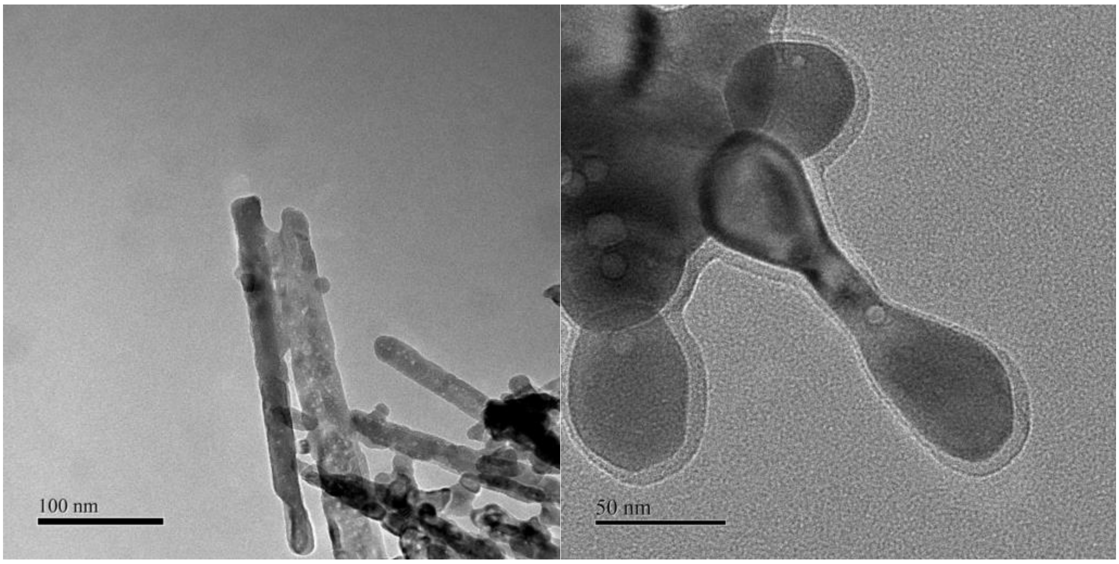

Figure SI-8. Comparison of two heating strategies of CeO2 nanorods:
Left: suspension of powder on Si3N4 TEM grid prior to furnace heating in air up to 950° C, and
right: suspension of powder onto standard carbon film after furnace heating in air up to 950° C.

Simulation Rationale and Supplementary Computational Figures:

Most simulations proceed by generating a model structure, which is then used to predict properties. For example, one might generate a model of a nanorod using crystallographic data and manually introduce voids into the rods as seen experimentally.
However, real ceria nanorods are never pristine; rather they likely comprise a rich microstructure, such as polar CeO_2(100) and non-polar CeO_2 (111) surfaces, edges where two surfaces meet, surface steps and corners together with point and extended defects. Such structural features are metastable and intrinsically of high energy compared to the perfect, pristine nanorod.
We postulate that voids emanate from high-energy microstructural 'active sites' because the activation energy barriers associated with their evolution is likely lower from a high-energy state compared to evolution from the thermodynamically low energy structural configurations associated with the pristine parent material.
To increase the probability of simulation being able to 'observe' (predict) mechanisms underpinning void formation, one must capture this rich microstructure within the model. However, it is very difficult to 'manually' introduce a complex microstructure that accord with the real material within a single model. Here, we achieve this by simulating, in part, the experimental method of nanorod fabrication. In particular, real ceria nanorods emanate from some kind of crystallisation process during synthesis and it is this process that endows nanorods with rich microstructures. Accordingly, we generate atom level models of nanorods by simulating crystallisation using methods we developed previously [1,2]

Generating Atomistic Models

A nanoparticle of CeO_2 comprising 15,972 atoms (5324 Ce, 10648 O) was melted at 8000K using Molecular Dynamics simulation, MD, and cooled to 3750K. The nanoparticle was then placed in a simulation box, while imposing periodic boundary conditions. One of the dimensions of the box was then reduced to enable cluster-cluster interactions along one direction. Under MD simulation, performed at 3750K, the nanoparticle agglomerated with its (periodic) neighbour to form an amorphous nanorod.
Starting from this amorphous nanorod precursor, MD simulation within an NVT ensemble (constant Number of atoms, Volume and Temperature) was performed for 2 ns at 3400K. At a particular instant in time a crystalline seed spontaneously evolved within the amorphous sea of Ce and O ions. This seed then propagates the crystallisation of the rod as amorphous ions, at the crystalline-amorphous interface, move to lattice positions on the surface of the seed. The model resulting from this procedure is shown in fig. SI-7 and has a [211] growth direction.
A second simulation was performed, starting from the amorphous rod precursor, and MD simulation performed for 4 ns at 3750 K. This procedure resulted in a nanorod with [110] growth direction, fig SI-10.

Validation

To have confidence in using the models to simulate directly the embryonic stages of void formation and unravel their mechanistic underpinnings, we looked to see how closely the model nanorods accord with experiment.
Perhaps most important is that simulated crystallisation, starting from an amorphous precursor, led to the formation of crystalline ceria nanorods with [211] and [110] growth

directions. Our experiments showed real ceria nanorods with both [211] and [110] growth directions, fig 2. We note that the model nanostructures were not created artificially, by imposing a crystallographic growth direction and crystal structure; rather, analogous to experiment, they crystallised 'naturally' and spontaneously. The only variable that can be changed for these simulations is temperature.

In the main text, describing the real nanorods, we stated that they exhibit non-regular hexagon cross-section, enclosed by {111}/{110} facets for <211> rod axis or {100}/{110} facets for <110> axis. Our simulations can provide further insight into these morphologies, which is particularly important because these microstructural features will likely influence (either promote or hinder) the evolution of voids. Our models reveal non-regular hexagonal cross-section for both nanorods, SI-9, SI-10, in accord with our experiments. For the ceria nanorod with [211] growth direction, fig SI-9, we observe {111} and {100} facets and small domains of {110}. CeO_2(100) is intrinsically unstable because it is a dipolar surface [3] and we observe significant structural rearrangement that occurs to quench the dipole. In particular, half the oxygen is missing from the outermost atomic layer to help eliminate the dipole. Our models accord with structures observed experimentally using HRTEM [4]

The model for a ceria nanorod with [110] growth direction is shown in fig SI-8. This nanorod is bounded by {111} and {100} surfaces together with surface steps on both {111} and {100} surfaces. The structure of the steps accord well with step structures measured experimentally [5,6]. Specifically, experiment shows that "the (111) surface exhibits alternating steps having (110) and (001) facets." Our models reveal steps on (111) surfaces with (110) facets, fig SI-9(b) and fig SI-11, together with (001) facets, fig SI-9(a) and SI-10(a). We also observe substantial faceting into domains of {111}. This is because ceria {111} surfaces are more stable than either {100} or {110} surfaces.

In summary, we have 'simulated synthesis', using MD, to capture the crystallisation, which evolves a thermodynamically stable crystal structure. In particular, our models reveal:

- (Fluorite) crystal structure of ceria nanorods.
- Ceria nanorods with [110] and [211] growth directions.
- Nanorods with {111}, {110} and {100} surfaces.
- Structural rearrangements to quench the dipole associated with {100} surfaces.

All these structural features are observed in real ceria nanorods. In particular, that simulation is able to capture these features, without any *a priori* information (i.e. starting from an amorphous precursor) by simulating both the kinetics and thermodynamics of the non-equilibrium crystallisation processes that operate, provides strong evidence of its capability. Accordingly, we can have confidence in using the model to simulate processes, such as void evolution and properties including changes in catalytic activity.

Void Evolution Mechanism

The images presented, figs SI 9-12, represent low-temperature model structures of the ceria nanorods. The models were then used to simulate (experimental) annealing by performing MD simulation at high temperature. Analysis of the MD trajectories reveals substantial mobility of both Ce and O ions on {100} and {110} surfaces, steps, edges and corners. In comparison, little ionic mobility was observed on {111} plateau regions. An animation showing the mobility of oxygen (coloured red) and cerium (coloured blue) on the surface of a ceria nanorod is deposited as SI.

Ionic mobility is associated with atoms moving off their lattice sites creating vacancies. The vacancy is able to migrate deeper within the nanorod when subsurface atoms migrate to

the surface to fill surface vacancies. The mobility is vacancy driven and we observed concerted motion analogous to that we observed previously in BaF_2 and CaF_2, which also conform to the fluorite structure [7].
Preferential migration of ions on {100}, {110} surfaces and surface steps, compared to {111}, to form a vacancy is expected because the energy required to form a vacancy on {111} surfaces is higher than on {100}, {110} and stepped sites [8].
Additional simulations reveal that ionic mobility increases when cerium is reduced to Ce^{3+} (together with charge compensating oxygen vacancies). This accords with experiment, which showed that cationic mobility on ceria {100} surfaces is higher when the surface is reduced compared to when it is fully oxidised [4]. This work will be presented in more detail in a later paper.

In summary, our simulations predict that

- Ce and O vacancies evolve spontaneously on high-energy ceria nanorod surfaces and steps.
- Vacancy formation is expediated when Ce^{4+} is reduced to Ce^{3+}.
- Ce and O vacancies diffuse deeper into the nanorod via a concerted mechanism.
- Ce and O vacancies in the nanorod agglomerate to form larger voids.
- Voids in the model evolve into polyhedral morphologies with {111} surfaces truncated by {100} and {110} (internal surfaces) – observed in our experiments in this study.
- {111} (internal) surfaces of voids in the model nanorod align with {111} (external) surfaces of the nanorod – observed in our experiments.

Such close accord with experiment increases confidence that the simulation is able to predict the mechanism of void evolution within the rods, which is difficult to elucidate experimentally. Specifically, we predict that, under annealing conditions, voids in ceria nanorods, via spontaneous vacancy formation on high-energy surfaces. The vacancies then diffuse into the nanorod, and agglomerate into voids.

Outlook

In addition to modelling void evolution, we predict a change in catalytic activity as a consequence of the voids. Specifically, we find that the energy required to extract oxygen from the surface, to participate in an oxidative catalytic reaction, is influenced by the presence of the voids, fig 9. Previously, simulation showed that surface stability and vacancy energies are directly linked with catalytic activity [8] and helped establish the concept of 'reactive surfaces'. Although the paper was published over 25 years ago it shows that simulation is able to reliably provide fundamental scientific insight that has remained relevant and insightful today. More recently, Aryanpour and co-workers showed [9] that the "energy for oxygen vacancy formation is a simple yet powerful activity descriptor." We advocate that it is reasonable to use such methods to predict that voids confer catalytic activity *changes* upon nanorods. Accordingly, mechanistic insight and understanding of void evolution will enable their control, facilitating tuneable properties via structural engineering.

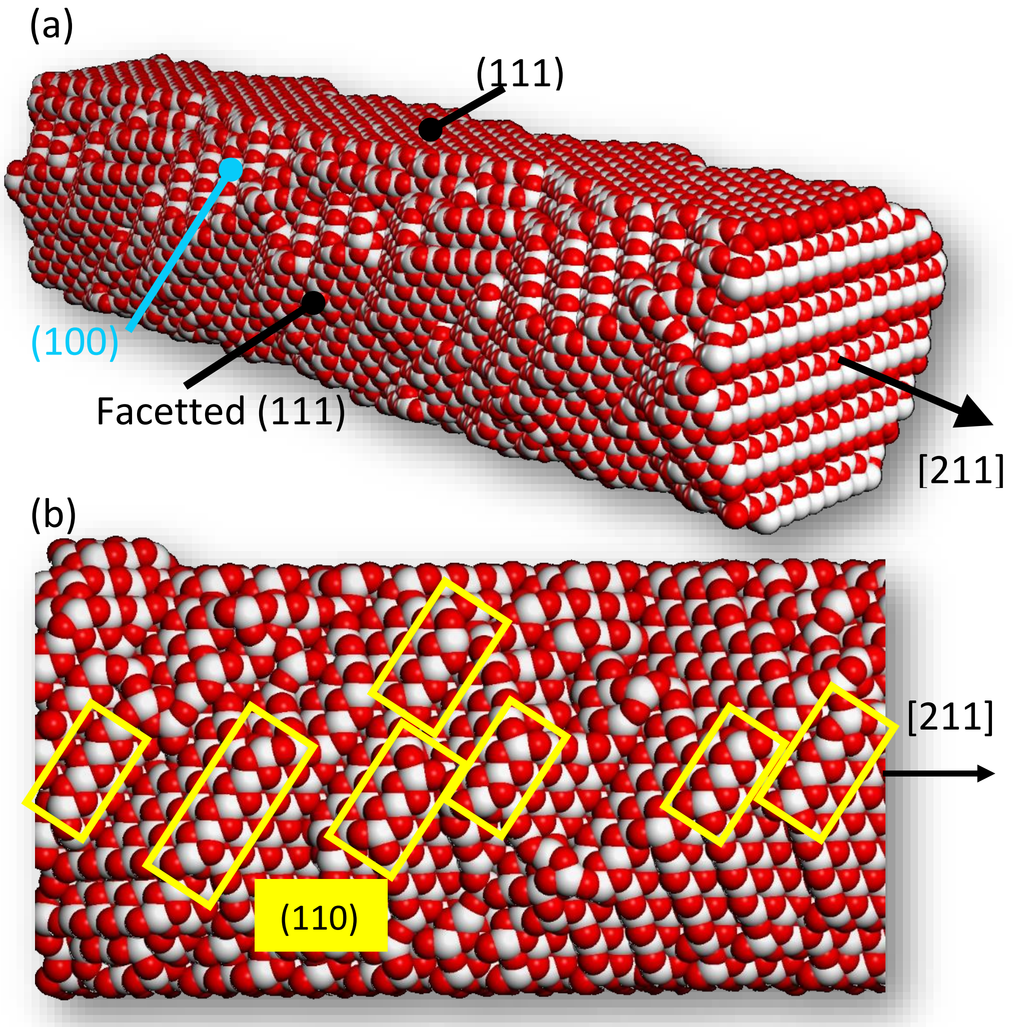

Fig SI-9 Atom level model of a ceria nanorod with [211] growth direction. (a) perspective view showing distorted hexagonal cross section, (111) and (100) surfaces together with surfaces that have facetted into {111}. (b) side view of the nanorod showing more clearly domains of {110}. Domains of {110} can perhaps be better described as representing the edges between facetted {111} surfaces.

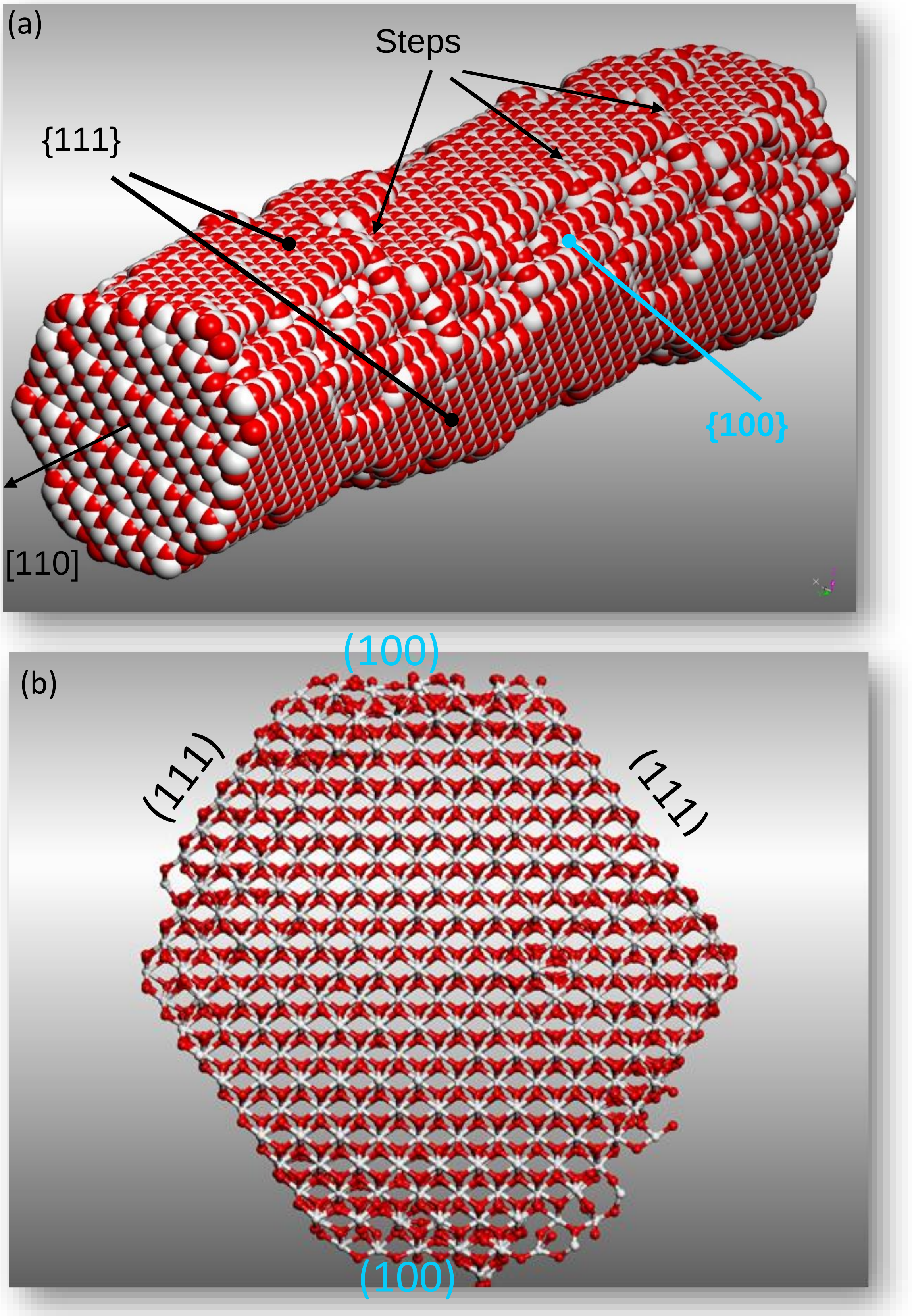

Fig SI-10 Atom level model of a ceria nanorod with [110] growth direction. (a) perspective view showing, {111} and {100} surfaces together with steps on these surfaces. (b) cross section of the rod showing a distorted hexagonal profile and {111} and {100} surfaces.

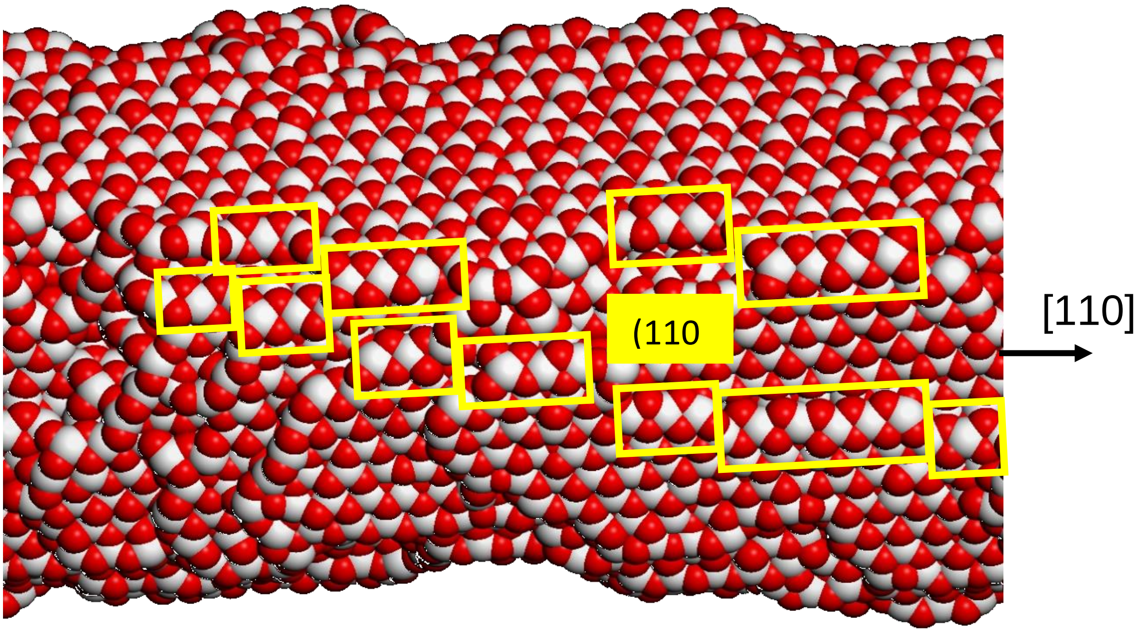

Fig SI-11 Side view of the ceria nanorod with [110] growth direction showing domains of {110}. We note that these domains may also be considered as steps on the {111} surfaces. The image is viewed along <110>.

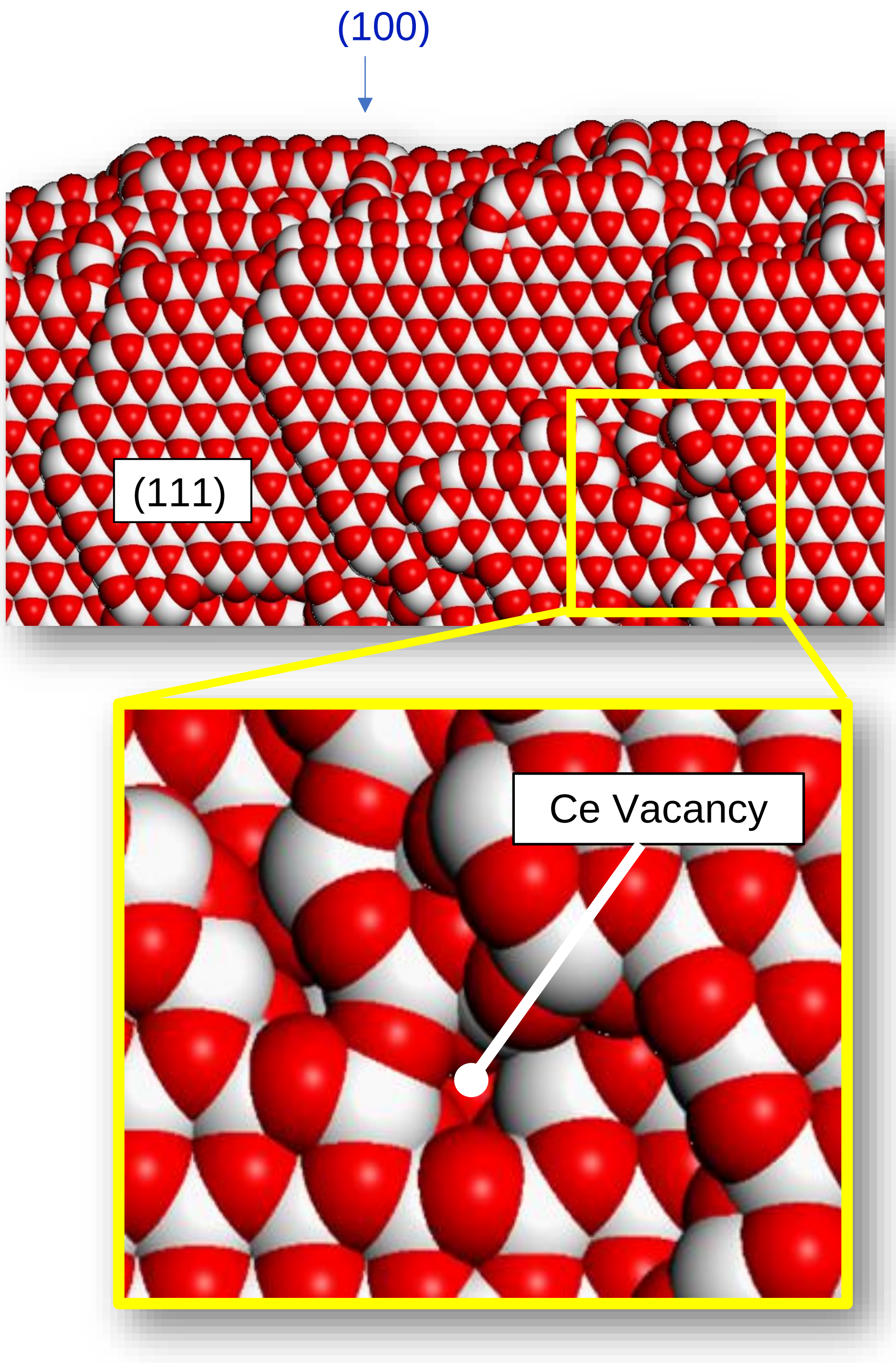

Fig SI-12 Image of a model ceria nanorod, with [110] growth direction. (a) viewed along <111> showing steps on the (111) surface. (b) Enlarged segment of (a) showing a Ce vacancy that has evolved close to a step.

References

1 Sayle, D. C.; Feng, X.; Ding, Y.; Wang, Z. L.; Sayle, T. X. T.
"Simulating Synthesis": Ceria Nanosphere Self-Assembly into Nanorods and Framework Architectures.
J. Am. Chem. Soc. **2007**, *129* (25), 7924–7935. https://doi.org/10.1021/ja070893w

2 Sayle, D. C.; Seal, S.; Wang⊥, Z. W.; Mangili, B. C.; Price, D. W.; Karakoti, A. S.; Kuchibhatla, S. V. T. N.; Hao, Q.; Möbus, G.; Xu, X. and Sayle, T. X. T.
Mapping Nanostructure: A Systematic Enumeration of Nanomaterials by Assembling Nanobuilding Blocks at Crystallographic Positions.
ACS Nano **2008**, 2, 6, 1237–1251. https://doi.org/10.1021/nn800065g

3 Goniakowski, J.; Finocchi, F. and Noguera, C.
Polarity of oxide surfaces and nanostructures
Rep. Prog. Phys. **2007**, 71 016501 https://iopscience.iop.org/article/10.1088/0034-4885/71/1/016501

4 Bugnet, M.; Overbury, S. H.; Wu, Z. L. and Epicier, T.
Direct Visualization and Control of Atomic Mobility at {100} Surfaces of Ceria in the Environmental Transmission Electron Microscope
Nano Lett. **2017**, 17, 12, 7652–7658 https://doi.org/10.1021/acs.nanolett.7b03680

5 Gritschneder, S.; Namai, Y.; Iwasawa, Y. and Reichling, M.
Structural features of CeO2(111) revealed by dynamic SFM
Nanotechnology, **2005**, 16, S41 http://dx.doi.org/10.1088/0957-4484/16/3/008

6 Torbrügge, S.; Cranney, M. and Reichlinga, M.
Morphology of step structures on CeO2(111)
Appl. Phys. Lett. **2008**, 93, 073112 https://doi.org/10.1063/1.2969790

7 Düvel, A.; Heitjans, P.; Fedorov, P.; Scholz, G.; Cibin, G.; Chadwick, A. V.; Pickup⊥, D. M.; Ramos, S.; Sayle, L. W. L.; Sayle, E. K. L.; Sayle, T. X. T. and Sayle, D. C.
Is Geometric Frustration-Induced Disorder a Recipe for High Ionic Conductivity?
J. Am. Chem. Soc. **2017**, 139, 16, 5842–5848 https://doi.org/10.1021/jacs.7b00502

8 Sayle, T. X. T.; Parker, S. C. and Catlow, C. R. A.
The role of oxygen vacancies on ceria surfaces in the oxidation of carbon monoxide
Surf. Sci., **1994**, 316, 329-336; https://doi.org/10.1016/0039-6028(94)91225-4
Conesa, J. C.
Computer modelling of surfaces and defects on cerium dioxide.
Surf. Sci., **1995**, 339, 337-352. https://doi.org/10.1016/0039-6028(95)00595-1

9 Aryanpour, M.; Khetan, A. and Pitsch, H.
Activity Descriptor for Catalytic Reactions on Doped Cerium Oxide
ACS Catal. **2013**, 3, 6, 1253–1262. https://doi.org/10.1021/cs400034c
